# A Highly Sensitive Deep-Sea Hydrodynamic Pressure Sensor Inspired by Fish Lateral Line

**DOI:** 10.3390/biomimetics9030190

**Published:** 2024-03-20

**Authors:** Xiaohe Hu, Zhiqiang Ma, Zheng Gong, Fuqun Zhao, Sheng Guo, Deyuan Zhang, Yonggang Jiang

**Affiliations:** 1 School of Mechanical, Electronic and Control Engineering, Beijing Jiaotong University, Beijing 100044, China; xhhu@bjtu.edu.cn (X.H.); fqzhao@buaa.edu.cn (F.Z.); shguo@bjtu.edu.cn (S.G.); 2 School of Mechanical Engineering and Automation, Beihang University, Beijing 100191, China; zhenggong@buaa.edu.cn (Z.G.); zhangdy@buaa.edu.cn (D.Z.); 3 Department of Biomedical Engineering, City University of Hong Kong, 83 Tat Chee Avenue, Kowloon, Hong Kong 999077, China

**Keywords:** lateral line, biomimetic, deep-sea, hydrodynamic pressure sensor, piezoelectric nanofiber mat

## Abstract

Hydrodynamic pressure sensors offer an auxiliary approach for ocean exploration by unmanned underwater vehicles (UUVs). However, existing hydrodynamic pressure sensors often lack the ability to monitor subtle hydrodynamic stimuli in deep-sea environments. In this study, we present the development of a deep-sea hydrodynamic pressure sensor (DSHPS) capable of operating over a wide range of water depths while maintaining exceptional hydrodynamic sensing performance. The DSHPS device was systematically optimized by considering factors such as piezoelectric polyvinylidene fluoride–trifluoroethylene/barium titanate [P(VDF-TrFE)/BTO] nanofibers, electrode configurations, sensing element dimensions, integrated circuits, and packaging strategies. The optimized DSHPS exhibited a remarkable pressure gradient response, achieving a minimum pressure difference detection capability of approximately 0.11 Pa. Additionally, the DSHPS demonstrated outstanding performance in the spatial positioning of dipole sources, which was elucidated through theoretical charge modeling and fluid–structure interaction (FSI) simulations. Furthermore, the integration of a high Young’s modulus packaging strategy inspired by fish skull morphology ensured reliable sensing capabilities of the DSHPS even at depths of 1000 m in the deep sea. The DSHPS also exhibited consistent and reproducible positioning performance for subtle hydrodynamic stimulus sources across this wide range of water depths. We envision that the development of the DSHPS not only enhances our understanding of the evolutionary aspects of deep-sea canal lateral lines but also paves the way for the advancement of artificial hydrodynamic pressure sensors.

## 1. Introduction

Despite the vast abundance of resources in the ocean, our understanding of it, particularly the deep sea, remains limited. Unmanned underwater vehicles (UUVs) have emerged as crucial tools in the field of ocean exploration [1,2,3]. These vehicles have proven instrumental in various activities such as resource discovery, environmental monitoring, rescue operations, and archaeological research [4,5,6]. With their operational capabilities spanning a wide range, UUVs are capable of exploring environments ranging from shallow waters to the depths of the deep sea [7]. UUVs primarily employ acoustic and vision-based techniques to explore their surroundings. However, these methods have exhibited certain limitations, including potential harm to marine creatures and reduced effectiveness in murky or dark waters [8,9,10]. In contrast, the hydrodynamic sensing strategy offers distinct advantages, as it is capable of operating in diverse water conditions while remaining environmentally friendly, setting it apart from traditional approaches [11,12,13,14]. Thanks to the rapid advancements in flexible electronics, researchers have successfully manufactured a range of deep-sea pressure sensors [15,16]. These sensors have demonstrated the capability to assess sea depths up to 2000 m. Nevertheless, the accurate detection of subtle hydrodynamic changes in the deep sea remains a significant and ongoing challenge.

Learning from nature is an effective and reliable approach to developing kinds of artificial devices that are applied in engineering fields [17,18]. Over thousands of years of evolution, fish have developed a remarkable mechanosensory lateral line system that enables them to detect hydrodynamic stimuli and navigate their surroundings, even in challenging conditions such as muddy water and deep-sea darkness. This lateral system consists of numerous specialized sensing elements known as neuromasts, which can be categorized into two types, i.e., superficial neuromasts (SNs) and canal neuromasts (CNs). SNs, positioned on the skin surface, are highly sensitive to flow velocity. Meanwhile, CNs located within fluid-filled sub-epidermal canals, detect pressure gradients between adjacent canal pores. Furthermore, the canals themselves play a crucial role in filtering out external noise [19].

Taking inspiration from the remarkable sensitivity of a fish’s lateral line system, engineers have successfully developed various types of canal artificial lateral line (CALL) systems by employing diverse sensing mechanisms [20,21,22]. Among these mechanisms, the piezoelectric working principle offers specific advantages in dynamic stimulus detection and fast responses. For instance, Asadnia et al. constructed a CALL system by integrating a PZT piezoelectric diaphragm-based pressure sensor into PDMS microchannels [23]. The results demonstrated that the integrated canal effectively attenuated low-frequency signals below 10 Hz, mimicking the natural filtering capability of a fish’s canal lateral line system. Additionally, Sharif et al. developed a CALL system by incorporating an ionic-polymer metal composite (IPMC)-based cilium sensor into a 3D-printed canal filled with a viscous fluid [24]. The developed sensor was successfully validated for its ability to detect pressure differences between the canal pores, with the sensing performance being influenced by the properties of the canal fluids. To further enhance the capabilities of CALL devices, Jiang et al. have developed a series of hydrodynamic pressure sensors by integrating piezoelectric sensing microcantilevers with PDMS microchannels. These devices include PVDF film-based sensors [25], P(VDF-TrFE) nanofiber mat-based sensors [26], and P(VDF-TrFE)/BTO nanofiber mat-based sensors [27]. Through careful design of the device’s structure and materials, the detection limit of the sensor has been significantly improved from 0.11 Pa to 3.2 mPa, as expected. However, these existing works have several limitations, which are outlined as follows: (1) The developed sensor necessitates an additional large-volume charger amplification instrument for signal collection, which is susceptible to environmental noise interference. (2) The hydrodynamic sensing performance of the developed devices has not been evaluated in deep-sea environments, potentially due to inadequate structural design or material selection.

Natural evolution has favored the survival of various organisms in the demanding conditions of the deep sea, including fishes, krill, and other species [28]. Deep-sea fish, in particular, have undergone significant adaptations to their lateral line systems, demonstrating several characteristics associated with deep-sea life. These adaptations include enlarged SNs, concentrated lateral line canals, and other notable features [28]. For example, Marsha et al. found that deep-sea fish species such as Poromitru cupito and Melanonus zugmayeri, residing in the meso-bathypelagic boundary, possessed widened head canals, a lack of body canals, and an extensive arrangement of SNs. This cephalic lateral line canal system exhibits robust development, likely due to the need to withstand high pressures in deep-sea environments while ensuring normal mechanosensory performance [29]. Additionally, many deep-sea organisms have evolved rigid shells to thrive in the harsh conditions of their habitats [30,31].

Taking inspiration from the cephalic canal lateral line system found in deep-sea fish, we presented the development of a deep-sea hydrodynamic pressure sensor (DSHPS) capable of operating across a wide range of water depths while maintaining exceptional hydrodynamic sensing performance. The key features of the proposed DSHPS include a piezoelectric microcantilever-based sensing element, an integrated pre-amplification circuit, and a high Young’s modulus package inspired by the fish skull. Through systematic optimization encompassing aspects such as piezoelectric polyvinylidene fluoride–trifluoroethylene/barium titanate [P(VDF-TrFE)/BTO] nanofibers, electrode configurations, sensing element dimensions, integrated circuits, and packaging strategies, the DSHPS achieved remarkable pressure gradient response. Notably, it demonstrated a minimum pressure difference detection capability of approximately 0.11 Pa. In addition to investigating the pressure gradient response, we successfully evaluated the performance of the DSHPS in the spatial positioning of dipole sources. The spatial positioning mechanism employed by the DSHPS was elucidated and verified through theoretical charge modeling and fluid–structure interaction (FSI) simulations. Furthermore, we assessed the hydrodynamic sensing capabilities of the DSHPS in various hydrodynamic environments at different water depths, including 0 m, 500 m, and 1000 m. As anticipated, the developed DSHPS consistently maintained exceptional hydrodynamic sensing performance, even when transitioning from shallow waters to deep-sea environments. Moreover, the DSHPS demonstrated reliable and reproducible positioning performance for subtle hydrodynamic stimulus sources across this wide range of water depths. We believe that the development of the DSHPS not only enhances our understanding of the evolutionary aspects of deep-sea canal lateral lines but also contributes to the advancement of artificial hydrodynamic pressure sensors.

This paper is organized as follows: In the Section 2, we introduced the design and development of the DSHPS, the characterization pre-amplification circuit evaluation, the dipole source experimental platform validation, the hydrodynamic sensing performance evaluation of the DSHPS, and the FEA of the DSHPS. In the Section 3, we presented the optimization of the DSHPS in terms of the materials, structures, dimensions, and packaging strategy. We then exhibited the fabrication results, carried out experiments on the hydrodynamic sensing performance of the DSHPS in a shallow water environment, performed an object localization performance of the DSHPS, and examined the hydrodynamic sensing performance of the DSHPS in a deep-sea environment. In the Section 4, the design and performance of the DSHPS were summarized.

## 2. Materials and Methods

### 2.1. Design and Development of DSHPS

**Figure 1:**
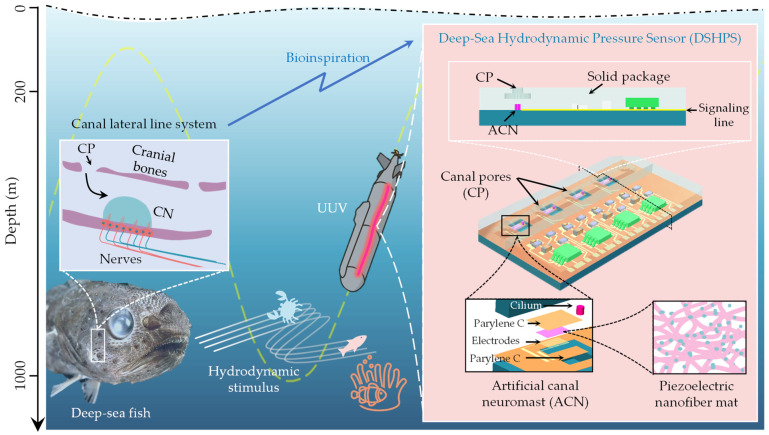
Schematic illustration of the proposed DSHPS, inspired by the evolved canal lateral line system in deep-sea fish.

Taking inspiration from the canal lateral line system observed in deep-sea fish, we proposed a deep-sea hydrodynamic pressure sensor (DSHPS) to detect subtle hydrodynamic stimuli in deep-sea environments (Figure 1). The DSHPS comprised four microcantilever-based sensing elements mimicking the canal neuromast. The microcantilever is constructed using a Parylene C substrate, interdigital electrodes, a piezoelectric P(VDF-TrFE)/BTO nanofiber mat, and a biomimetic cilium located at the end of each cantilever. Similar to the fish lateral line prototype, these sensing elements were situated in microchannels that connect to the surrounding environment through a series of canal pores.

The pressure difference between adjacent canal pores induces fluid movement inside the microchannel, resulting in the deflection of the microcantilever. Consequently, the generated mechanical stress on the microcantilever is converted into electric signals by the piezoelectric nanofiber mats. To enhance its anti-noise capability, a pre-amplifier circuit is integrated with the DSHPS. The skulls of deep-sea fish are typically composed of dense, lightweight, and strong bone tissue that is well-suited for supporting the fish’s facial features and protecting its brain. These bone tissues often exhibit high mechanical properties, including a high Young’s modulus. Drawing insights from the cephalic canal lateral line system observed in deep-sea fish, we proposed a high Young’s modulus packaging strategy to ensure the perception performance of the DSHPS even at depths of 1000 m in the deep sea.

**Figure 2:**
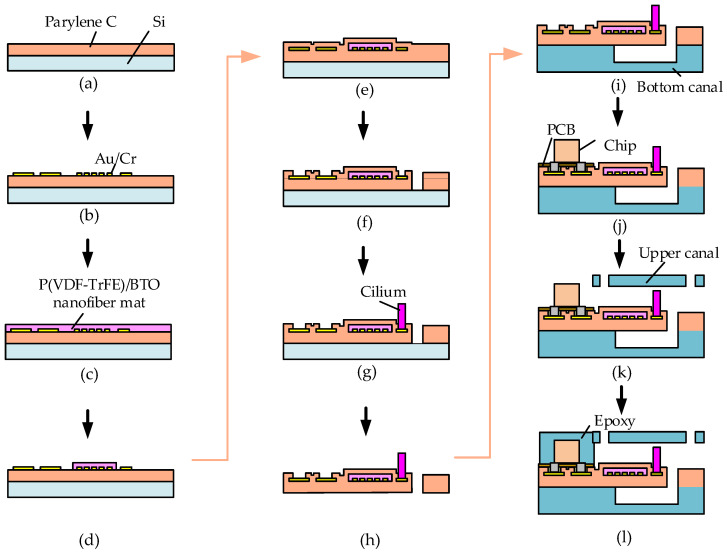
Fabrication process of the DSHPS. (**a**) Deposition of a Parylene C layer on a silicon wafer; (**b**) Forming the Au/Cr interdigital electrodes and pad patterns; (**c**) Electrospun a layer of P(VDF-TrFE)/BTO nanofiber mat; (**d**) Patterning the P(VDF-TrFE)/BTO nanofiber mat by reactive ion etching technology; (**e**) Deposition of a Parylene C layer; (**f**) Etching the Parylene C layer to form the cantilever and expose the pad patterns; (**g**) Attaching the SU-8 cilia; (**h**) Separating the integrated Parylene C structure from the silicon wafer; (**i**) Combining the Parylene C layer with the bottom canal; (**j**) Bonding the PCB to the pad on the Parylene C layer; (**k**) Installing the upper canal; (**l**) Encapsulating the circuit components with a layer of epoxy resin.

Using MEMS (Microelectromechanical Systems) techniques, we successfully developed the proposed DSHPS and the fabrication process is illustrated in Figure 2. Initially, a silicon wafer underwent baking in a hot oven at 110 °C for 15 min to generate an oxide layer. Subsequently, a 12 μm thick Parylene C layer was deposited onto the silicon wafer via vacuum deposition (PDS-2010, Specialty Coating System, Indianapolis, IN, USA). Interdigital electrodes of Au/Cr (160 nm/40 nm) were patterned on the Parylene C using photolithography, with a photoresist (AZ4620) serving as the mask and employing a magnetron sputtering deposition process. A 20 μm P(VDF-TrFE)/BTO nanofiber mat, serving as the sensing element, was electrospun onto the Au/Cr layer using a far-field electrospinning process [32]. During the electrospinning process, a 22 G syringe needle with an inner diameter of 0.4 mm and an outer diameter of 0.7 mm was utilized. The distance between the needle tip and the substrate was maintained at 15 cm. The needle was subjected to a high pressure of 12 kV, with a solution feed rate of 1 mL/h. The excess P(VDF-TrFE)/BTO nanofibers extending beyond the rectangular area of the cantilever were selectively etched using a reactive ion etching (RIE) process with a shadow mask. Subsequently, a 6 μm Parylene C protective layer was deposited at the top of the P(VDF-TrFE)/BTO nanofiber mat. The cantilever and pad patterns with regular shapes were defined using RIE technology. The P(VDF-TrFE)/BTO nanofiber mat was then poled by subjecting it to an electric field of 10 V/µm for 1 h at 80 °C using a micromanipulation probe station. Following that, SU-8 cilium, fabricated through photolithography processes, was securely attached to the ends of the cantilevers using epoxy resin. The lower and upper canal structures were produced using 3D printing technology. The Parylene C substrate was separated from the silicon wafer and subsequently bonded to the lower substrate using epoxy resin.

The circuits were fabricated using PCB processing techniques, utilizing copper plating as the conductive layer. The final dimensions of the PCB circuit board measured 38 mm × 12 mm × 100 μm. For the electronic components of the circuit section, small-package surface-mounted components were chosen. Low-temperature soldering paste was employed to bond and establish electrical connections between all the components, including the operational amplifiers (AD8602), capacitors, resistors, and their corresponding contact pads on the Cu/PI substrate. The solder pads on the circuit board were electrically linked to the pins on the bottom Parylene C layer using a conductive silver paste, which was then secured with epoxy resin. The conductive silver glue (H20E, EPOTEK, Billerica, MA, USA) was cured at a temperature of 60 °C for a duration of 4 h. The upper canal structure was subsequently secured in place using epoxy resin. To safeguard the circuit against high water pressure, a layer of epoxy resin was applied to encapsulate the circuit components.

### 2.2. Characterization

Scanning electron microscopy (SEM, JSM-5800, JEOL, Akishima, Japan) and TEM (JEM-2100, JEOL, Japan) were used to collect surface morphology information of P(VDF-TrFE)/BTO nanofibers. The crystalline structures of the nanofibers were measured by XRD (D-max 2500, Rigaku, Tokyo, Japan, Cu Kα radiation, working voltage and current: 40 kV, 200 mA) and ATR-FTIR techniques (Nicolet 6700, Thermo Scientific, Waltham, MA, USA). PFM (MFP-3D, Asylum Research, Santa Barbara, CA, USA) was utilized to determine the piezoelectric properties of nanofibers.

### 2.3. Pre-Amplification Circuit Evaluation

A commercial PVDF sensing element (10 mm × 20 mm × 30 μm) encapsulated by PET film was used to evaluate the performance of the developed pre-amplification circuits. The experiment was conducted in a water tunnel with dimensions of 0.7 × 0.35 × 0.4 m^3^. A standard commercial charge amplifier (NEXUS Conditioning Amplifier-2692, Brüel & Kjær, Nærum, Denmark) with a transducer sensitivity of 10 pC/unit and an output sensitivity of 316 mV/unit was employed as a reference. A dipole (15 mm in diameter) driven by a pneumatic actuator was used as the incentive source. During the test, the distance between the PVDF and the dipole was kept at 10 mm, and the frequency of the dipole was 80 ± 3 Hz. The signals amplified by the developed pre-amplification circuits and commercial charge amplifier were collected by a data acquisition card (USB4711).

### 2.4. Dipole Source Experimental Platform Validation

The MS5401, with a sensitivity of 1.15 mV/Pa, was employed to calibrate the dipole source platform. A rigid plastic sphere with a diameter of 15 mm was used as the dipole, which was driven by a pneumatic actuator. The dipole and MS5401 were immersed in the water tunnel, which had dimensions of 0.7 × 0.35 × 0.4 m^3^, and was filled with water to a depth of 25 cm. The dipole vibrated perpendicularly to the MS5401 at a frequency of 80 ± 3 Hz. The pressure generated by the dipole was adjusted by changing the distance between the center of the dipole and the top surface of the MS5401. During the test, a DC-regulated power supply was used to provide ±2.5 V voltage. The output signal was filtered by a filter and then collected by a data acquisition card.

### 2.5. Hydrodynamic Sensing Performance Evaluation of DSHPS

#### 2.5.1. Shallow Water Experiment

Pressure gradient threshold detection: The test was conducted in the water tunnel with dimensions of 0.7 × 0.35 × 0.4 m^3^, filled with water to a depth of 25 cm. The dipole vibrated perpendicular to the canal at a frequency of 77 ± 3 Hz. The pressure field generated by the dipole could be adjusted by changing the distance between the center of the dipole and the top of the canal. During the test, a DC power supply was used to provide a voltage of ±2.5 V for the amplification circuit. After the output signal was filtered by the filter, it was collected by the data acquisition card, and the output signal was displayed in real-time through LabVIEW. Among them, the filters (3624, NF Electronic Instruments, Yokohama, Japan) filtering frequency was 55–95 Hz.

Vibrating source location testing: The test was conducted in the water tunnel, and the dipole vibrated perpendicularly to the DSHPS, with five positions corresponding to 15 mm and 37.5 mm directly above the five canal pores. During the experiment, a data acquisition card was used to simultaneously record the peak output voltages of the four sensing units.

#### 2.5.2. Deep-Sea Environment Test

The DSHPS was placed in the high-pressure vessel, and the pressure was supplied by a high-pressure nitrogen cylinder. The internal pressure was displayed on the pressure gauge in real-time. The frequency response characteristics of the DSHPS at positions 1 and 2 were tested at 0 Pa, 5 MPa, and 10 MPa, respectively. Position 1 (P1) refers to the paddles positioned directly above the canal pore, with the lower end of the blade located at a distance of 3 mm from the top surface of the DSHPS. Position 2 (P2) was situated 5 mm away from P1 along the rod’s direction. During the test, the DSHPS was placed on the platform inside the high-pressure vessel, and the vessel was filled with deionized water. The output signal was transmitted to the outside of the cavity through wires. The DC power supply provided DC voltage to the circuit, and the output signal was collected by a data acquisition card and the computer.

### 2.6. FEA of DSHPS

#### 2.6.1. Three-Dimensional FEA Modeling of Interdigital Electrodes

To gain more insight into the piezopotential distribution on the interdigital electrodes, FEM simulations were conducted using the coupled fluid–structure interaction (FSI) module of COMSOL Multiphysics by placing a sensing unit in a water canal. The dimension of the water canal was 15 mm × 3 mm × 1 mm, and the distance between adjacent canal pores was 7.5 mm, which was consistent with the DSHPS. A pressure difference of 0.21 Pa was applied to the canal pore.

#### 2.6.2. Resonant Frequency Simulation of the Cantilever

The resonant frequency of a cantilever was studied using COMSOL multiphysics simulation. The cantilever was composed of a Parylene C substrate, a P(VDF-TrFE)/BTO nanofiber mat, an SU-8 cilium, and a Parylene C protective layer. The dimensions of each part were consistent with the designed values. During the simulation, the P(VDF-TrFE)/BTO nanofiber mat was simplified to a uniform mat. One end of the cantilever beam was fixed. The Young’s modulus of the Parylene C, P(VDF-TrFE)/BTO nanofiber mat and SU-8 cilium were 2.2, 0.15 and 3.5 GPa.

#### 2.6.3. Deep-Sea High-Pressure Simulation

A 50 mm × 50 mm × 1000 m water area was used to generate 10 MPa pressure on the DSHPS. The dimensions of the DSHPS were consistent with the designed structure. The water density was 1000 kg/m^3^, and the gravitational acceleration was set to 9.8 m/s^2^. The Young’s modulus of the PCB and the welding pad were 2.5 and 3.6 GPa. For the simulation, the following assumptions were made for the circuits part: (1) rigid connections between the electronic components and PCB; (2) rigid connections between the flexible circuits and substrates, with close contact and no gaps; (3) structures were not affected by temperature. In the real world, the contact between electronic components, PCB, and substrate may not be completely rigid, which may affect the working stability of the DSHPS in deep-sea environments. In addition, the structure could also be affected by temperature, resulting in changes in performance.

#### 2.6.4. Vibrating Source Location Simulation

A two-dimensional FSI analysis model was used to simulate and analyze the dipole field in COMSOL Multiphysics. The dipole and the DSHPS were placed in a rectangular water tank with a size of 114 mm × 90 mm, and the length direction was about three times the length of the DSHPS. The diameter of the dipole was 15 mm, and the distance from the DSHPS to the dipole was D = 15 and 37.5 mm. The dipole vibrated perpendicular to the canal, and the vibration displacement was a sine wave with an amplitude of 1 mm and a frequency of 10 Hz. The dynamic viscosity coefficient of the water was set to 0.00101 Pa·s, and the density was 1000 kg/m^3^.

## 3. Results and Discussion

### 3.1. Material, Structural, Dimensional, and Package Optimization of DSHPS

**Figure 3:**
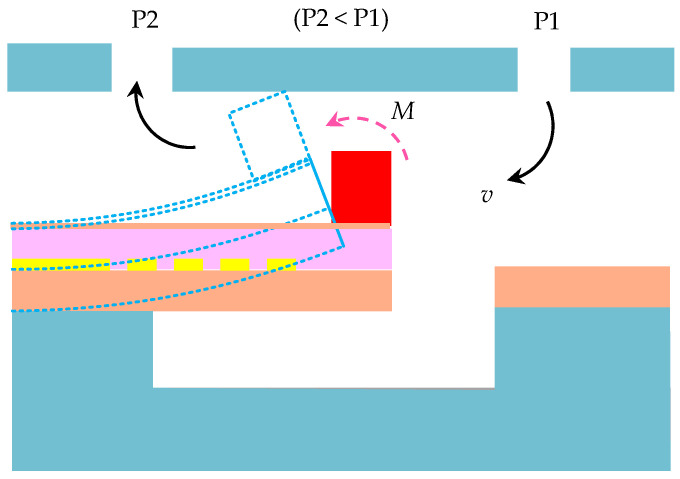
Schematic illustrating the operational mechanism of the proposed DSHPS.

In order to attain exceptional hydrodynamic sensing performance for the DSHPS, we conducted optimization in terms of the materials, structures, dimensions, and packaging strategy. The operational mechanism of the proposed DSHPS closely resembles that of the fish lateral line prototype, as depicted in Figure 3. Within the microfluidic canal, the fluid is propelled by the pressure difference between adjacent canal pores, generating a drag force on the pillar attached to the apex of the cantilever, resulting in the bending of the microcantilever. The piezoelectric nanofiber mats efficiently convert the mechanical stress experienced by the microcantilever into electrical signals.

The piezoelectric nanofiber mats have a direct influence on the hydrodynamic sensing performance of the DSHPS. The piezoelectric performance of the P(VDF-TrFE)/BTO nanofiber mat was optimized in our previous works from aspects of BTO content [32]. The P(VDF-TrFE)/BTO nanofiber mat fabricated with a BTO weight fraction of 5 wt % achieved the highest piezoelectric response.

**Figure 4:**
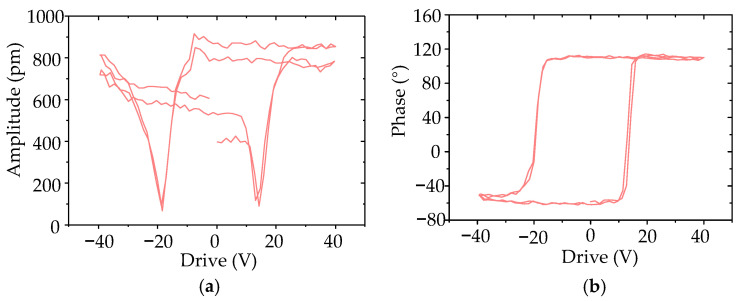
(**a**) Piezoresponse force microscopy (PFM) amplitude–voltage hysteresis loops of the fabricated P(VDF-TrFE)/BTO nanofiber; (**b**) Phase–voltage hysteresis loops of P(VDF-TrFE)/BTO nanofiber.

The crystallinity of the P(VDF-TrFE)/BTO nanofiber mat was assessed by X-Ray Diffraction (XRD) and Fourier transform infrared spectroscopy (FTIR) (Note S1, Figures S1 and S2). The prominent crystalline peak indicated the highly crystalline quality of the polar β-phase, which was as high as 81%. Furthermore, the piezoelectric constants *d*_33,eff_ were evaluated by piezoresponse force microscopy (PFM). Figure S3 shows the out-of-plane amplitude and phase images. The distinct contrast in the piezoresponse amplitude indicated the piezoelectric response of the P(VDF-TrFE)/BTO nanofibers. The bright (yellow) and dark (purple) contrast regions in the phase image manifest the domain as polarized in the upward and downward directions. The PFM hysteresis loops are shown in Figure 4; the characteristic butterfly-shape amplitude–voltage curves and near 180° contrast phase curves revealed that complete polarization switching occurred in the P(VDF-TrFE)/BTO nanofiber. The average *d*_33,eff_ of the P(VDF-TrFE)/BTO nanofiber was evaluated as approximately 44.75 pm/V.

**Figure 5:**
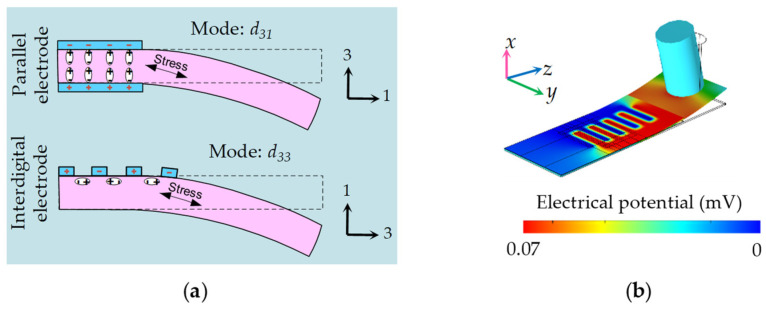
(**a**) Illustration demonstrating the optimization of the electrode configuration; (**b**) Three-dimensional finite element analysis (FEA) depicting the distribution of piezopotential between the interdigital electrodes.

The sensing performance of piezoelectric devices is significantly influenced not only by the choice of piezoelectric materials but also by the configuration of the electrodes. Piezoelectric films commonly operate in two working modes: *d*_31_ and *d*_33_ modes (Supplementary Note S2). However, in piezoelectric sensors with the same structure and stress conditions, the *d*_31_ mode is less efficient compared to the *d*_33_ mode. This discrepancy arises because the *d*_33_ parameter typically has twice the value of the *d*_31_ parameter [33,34]. To achieve the *d*_33_ working mode in the P(VDF-TrFE)/BTO nanofiber mat, we employed a combination of interdigital electrodes and cantilever structures, as depicted in Figure 5a. During the deformation of the piezoelectric cantilever, the internal stress aligns along the length of the cantilever (direction 1). In the case of common parallel electrodes, the polarization directions of the upper and lower electrodes are perpendicular to the length of the cantilever (direction 3). Consequently, the stress direction and the polarization direction are perpendicular to each other, corresponding to the *d*_31_ mode. However, the utilization of interdigital electrodes ensures that the polarization direction of the piezoelectric nanofiber mat aligns consistently with the stress direction, resulting in the *d*_33_ mode.

To visually elucidate the working mechanism of the proposed DSHPS device, transient electrostatic Multiphysics simulations were conducted. The piezopotential distribution of the P(VDF-TrFE)/BTO nanofiber mat with interdigital electrodes on the cantilever, induced by a pressure difference of 0.21 Pa between adjacent canal pores, was calculated (Figure 5b). The accompanying Movie S1 in the Supporting Information showcases this dynamic response process. On the Parylene C substrate, the P(VDF-TrFE)/BTO nanofiber mat exhibited a well-distributed piezopotential difference (∆V) of approximately 0.07 mV.

A theoretical response model was established (Supplementary Note S3 and Figure S4) to express the generated charge *Q* inside the DSHPS as follows:

(1) $$Q\propto t_{1}^{-n}DH^{2}{∆P}^{2}$$

where *n* is a positive value, *t*_1_ is the thickness of the P(VDF-TrFE)/BTO nanofiber mat, and *D* and *H* are the diameter and height of the cilium, respectively. The details are available in Supplementary Note S3 [35,36,37].

**Figure 6:**
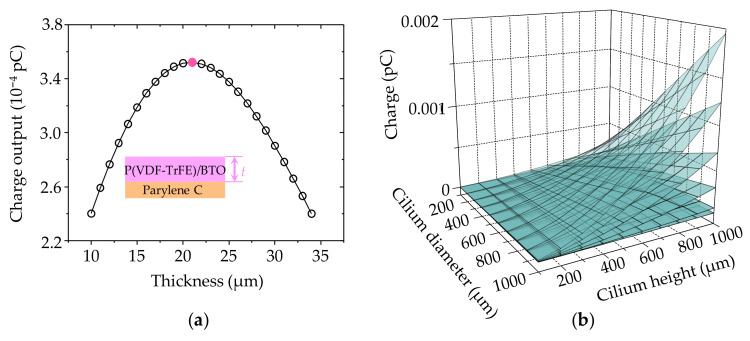
(**a**) Impact of the thickness of the piezoelectric P(VDF-TrFE)/BTO nanofiber mat on the mechanical sensing performance of the DSHPS; (**b**) Influence of cilium dimensions, including diameter (*D*) and height (*H*), on the sensing performance of the DSHPS under different hydrodynamic stimuli.

For the sake of processing convenience, a 12 μm thick Parylene C layer was employed. Based on the theoretical response model, optimization of the P(VDF-TrFE)/BTO nanofiber mat thickness and cilium dimensions can be achieved. As demonstrated in Figure 6a, the DSHPS exhibits the highest charge output when the thickness of the P(VDF-TrFE)/BTO nanofiber mat reaches 20 μm. Additionally, the impact of cilium dimensions on the sensing performance of the DSHPS was comprehensively investigated, as shown in Figure 6b. As anticipated, both the cilium height and diameter positively influence the performance of the DSHPS, owing to the increased fluid–structure interaction area. Considering the overall structure of the DSHPS, the cilium diameter was determined as 500 μm, while the cilium height was set as 600 μm.

The combination of interdigital electrodes and the piezoelectric sensing unit in the DSHPS can be treated as an equivalent parallel capacitor [38,39]. The total charge output of the interdigital electrodes can be considered as the cumulative sum of charges generated between each pair of interdigital electrodes. The width of each interdigital electrode and the distance between adjacent interdigital electrodes were set at 30 μm. With 14 pairs of interdigital electrodes employed in the DSHPS, the length of the microcantilever was determined as 2.4 mm. The width of the microcantilever was chosen as 1.2 mm, taking into account the canal structure. The microcantilever’s resonant frequency exhibited a prominent peak at the first resonant frequency of 793.9 Hz, which closely matched the theoretical value of 797.0 Hz (Figure S5).

**Figure 7:**
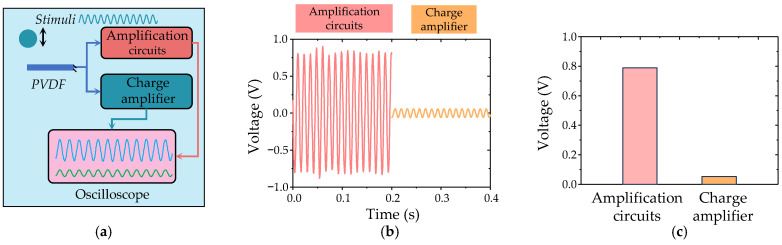
(**a**) Schematic diagram of the testing apparatus for characterizing the properties of the integrated circuit; (**b**,**c**) Performance evaluation of the circuit and the standard charge amplifier at an excitation frequency of 80 Hz.

A pre-amplification circuit was meticulously designed and developed, as depicted in Figures S6 and S7. To assess the performance of the developed amplification circuits, experimental evaluations were conducted using a commercial PVDF sensing element subjected to periodic stimulus, with a commercial charge amplifier serving as a benchmark for comparison (Figure 7a). As demonstrated in Figure 7b, the outputs obtained from the integrated circuits exhibited remarkable dynamic responses to the periodic mechanical stimulus, mirroring the behavior observed with the commercial charge amplifier. Notably, the integrated circuits showcased higher amplitude outputs compared to the commercial charge amplifier under the same mechanical stimulus (Figure 7c). Additionally, we conducted a comparison between the amplification times of the fabricated circuits and their corresponding theoretical values (Figure S8). The results demonstrate that the developed amplification circuits successfully fulfill their intended amplification function, with an amplification time of approximately 501 mV/pC.

In consideration of the DSHPS’s work in the deep-sea environment, the pressure resistance of the DSHPS is also an important factor that needs to be considered. To assess the ability of the proposed DSHPS to maintain optimal performance in deep-sea hydrodynamic environments, a comprehensive 3D fluid–structure interaction (FSI) analysis was performed using COMSOL Multiphysics. The DSHPS was subjected to a simulated water depth of 1000 m, and the mechanics experienced by the sensor were carefully examined (Figure S9). The material properties and dimensions utilized in the analysis were in alignment with the designed specifications of the DSHPS.

**Figure 8:**
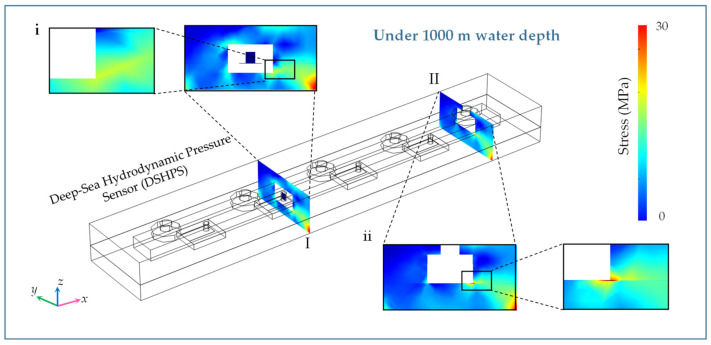
Stress distribution in the DSHPS with Epoxy canal at 1000 m deep-sea environments. (i) and (ii) represent the magnified views of section I and section II, respectively.

Given the structure of the DSHPS, two critical factors must be considered to ensure its reliability in deep-sea conditions: microchannel deformation or breakage and potential damage to the integrated circuits. The stress distribution within the epoxy and PDMS canal section is illustrated in Figure 8 and Figure S10. Notably, the Parylene C layer at the intersection of the upper and lower epoxy canals experiences the highest von Mises stress. The maximum von Mises stress is measured at 12 MPa, which falls within the yield strength range of Parylene C (55.16 MPa) [40]. Compared to the epoxy canal, significant deformation occurs in the PDMS canal. This observation confirms that the epoxy microchannel component is capable of functioning effectively in deep-sea environments.

**Figure 9:**
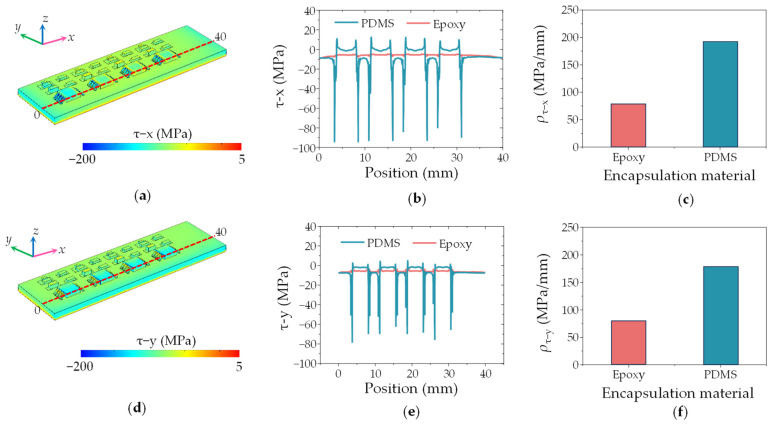
(**a**) Distribution of shear stress in the *x*-direction within the DSHPS; (**b**) Distribution of shear stress in the *x*-direction, and (**c**) shear stress density along the electric elements in the DSHPS; (**d**) Distribution of shear stress in the *y*-direction within the DSHPS; (**e**) Distribution of shear stress in the *y*-direction, and (**f**) shear stress density along the electric elements in the DSHPS.

The stress distribution of the circuit part packaged with PDMS and epoxy was studied. Figure 9a and d depict the shear stress distributed in the *x* and *y*-direction on the PCB substrate packaged by epoxy. For the PCB integrated with circuit components, peeling stress is the dominant stress that is responsible for the failure of the solder joints [41]. Compared with the PDMS package structure (Figure S11), the epoxy weakens the stress at the solder joints.

The distribution of *x* and *y*-direction shear stress along the length of 0 to 40 mm with PDMS and epoxy packaging is shown in Figure 9b,e. It can be seen that when the packaging material is epoxy, the shear stress values (9.7 MPa in the *x*-direction and 7.7 MPa in the *y*-direction) are smaller than that of PDMS (94.3 MPa in the *x*-direction and 78.0 MPa in the *y*-direction). The maximum peeling stresses in the outermost solder joint reaches 31 MPa [41]. Therefore, the PDMS-packaged DSHPS can cause damage to the solder joints. The stress density along electric elements in DSHPS is shown in Figure 9c,f. The stress density is smaller for epoxy resin than for PDMS. Therefore, choosing epoxy as the circuit packaging material can reduce the shear stress on the flexible substrate so that the electric elements can still work normally under 1000 m of static water pressure.

### 3.2. Fabrication Results

**Figure 10:**
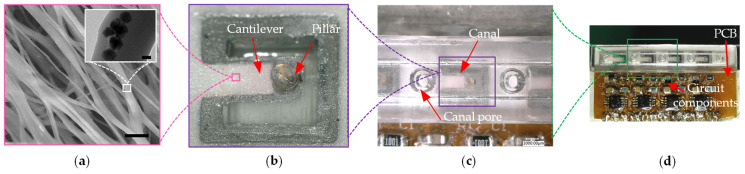
(**a**) Scanning electron microscopy (SEM) image of developed P(VDF-TrFE)/BTO nanofibers. The inset shows a transmission electron microscope (TEM) image of a single nanofiber with dark BTO particles. Scale bar: 5 μm; (**b**) Optical photograph of a sensing microcantilever integrated with a SU-8 cilium; (**c**) Optical image of an individual sensing element inside DSHPS; (**d**) Optical image of the developed DSHPS.

Consistent with expectations, Figure 10a illustrates the uniform distribution of the developed P(VDF-TrFE)/BTO nanofibers on the substrate, and the inset presents a transmission electron microscope (TEM) image showcasing a single nanofiber containing dark BTO nanoparticles. The fabricated sensing element exhibits a rectangular microcantilever structure, with the cilium firmly attached to the apex of the microcantilever, as depicted in Figure 10b. In Figure 10c, an optical photograph showcases the positioning of the sensing element between two adjacent canal pores. The completed DSHPS, featuring four sensing elements and integrated pre-amplification circuits, is presented in Figure 10d.

### 3.3. Hydrodynamic Sensing Performance of DSHPS in Shallow Water Environment

**Figure 11:**
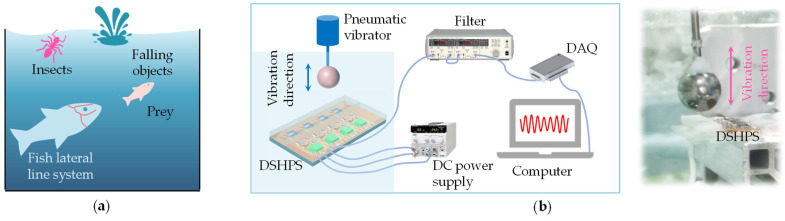
(**a**) Representative natural hydrodynamic stimuli perceived by the fish lateral line system; (**b**) Schematic illustration and optical image of the experimental platform used for evaluating the hydrodynamic performance of the DSHPS. The hydrodynamic stimulus is generated by a vibrating rigid sphere.

In nature, fish lateral line systems exhibit sensitivity to various types of disturbance stimuli, including droplets, swimming fish, and insects moving at the water’s surface (Figure 11a) [42,43]. In the laboratory setting, researchers simplify these hydrodynamic stimuli by generating a dipole field using a vibrating rigid sphere [27]. Inspired by this, we established an experimental platform to generate dipole fields for evaluating the hydrodynamic performance of the DSHPS. The platform consisted of a water tunnel with dimensions of 0.7 × 0.35 × 0.4 m^3^, filled with water to a depth of 25 cm, in which a rigid plastic sphere with a diameter of 15 mm was vibrated (Figure 11b). To validate the platform, we initially conducted experiments using a commercial MS5401 miniaturized pressure sensor (see Note S4 and Figure S12a). The sensitivity of the pressure sensor was calibrated to be 1.15 mV/Pa (Figure S12b). The hydrodynamic pressure experienced by the sensor decreased as the vertical distance between the dipole and the pressure sensor increased (Figure S12c). Additionally, the hydrodynamic pressure readings closely matched the theoretical values. These results confirmed that the experimental platform for generating dipole fields is functional and reliable.

To evaluate the hydrodynamic performance of the DSHPS, it was positioned horizontally at the center of the water tunnel, with the vibration direction of the dipole source perpendicular to the long axis of the DSHPS (Figure 11b). The pressure gradient perceived by the DSHPS can be mathematically expressed as follows [44]:

(2) $$\frac{dp}{dx}=\left(\frac{1}{l_{0}^{2}}-\frac{l_{0}}{{\left(l_{0}^{2}+{\left(\Delta x\right)}^{2}\right)}^{3/2}}\frac{\rho sr_{s}^{3}}{2\Delta x}\sin\left(2\pi ft\right)\right)$$

where *ρ* is the density of water, *r_s_* is the radius of the dipole, *f* and *s* are the vibration frequency (77 ± 3 Hz) and amplitude (1.1 mm), *l*_0_ is the distance between the dipole center and the sensor, and Δ*x* is the interval between adjacent canal pores (7.5 mm).

**Figure 12:**
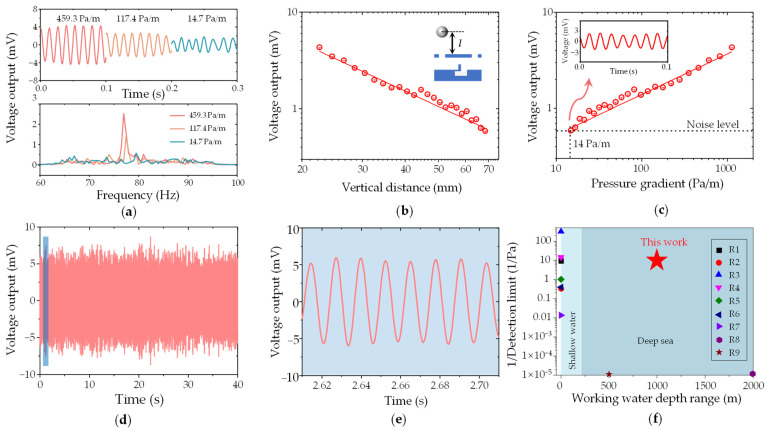
(**a**) Time-domain voltage output of the DSHPS and the corresponding fast Fourier transform (FFT) results in response to different hydrodynamic pressure gradients; (**b**) Vertical distance response of the DSHPS; (**c**) Pressure gradient response of the DSHPS; (**d**) Stability analysis of the DSHPS under a constant hydrodynamic pressure gradient of 1127.4 Pa/m for 3200 cycles (test frequency: 77 ± 3 Hz); (**e**) Zoomed-in plot in the time domain; (**f**) Performance comparison. The related references are available in Table S1 [15,16,25,27].

As depicted in Figure 12a, the DSHPS exhibited a remarkable dynamic response to the periodic stimulus generated by the dipole source. An increase in the hydrodynamic pressure gradient from 14.7 Pa/m to 459.3 Pa/m resulted in a corresponding increase in the amplitude of the DSHPS outputs. In terms of frequency domain outputs, the DSHPS demonstrated a characteristic frequency of 77 ± 3 Hz, which closely matched the excitation frequency of the dipole source.

The outputs of the DSHPS exhibited a decrease as the vertical distance increased from 22.5 to 68.5 mm (Figure 12b). This decrease can be attributed to the corresponding reduction in the hydrodynamic pressure gradient. The minimum pressure gradient detection threshold, defined as the pressure gradient corresponding to the noise level, was determined to be 14 Pa/m based on the pressure gradient response curve (Figure 12c). Considering an interval of 7.5 mm between adjacent canal pores, the DSHPS was capable of detecting a minimum pressure difference of approximately 0.11 Pa. A comparison of the voltage output between the simulation and experimental results of the proposed DSHPS device, responding to a series of pressure differences in shallow water environments, is shown in Figure S13. The voltage output of the experiment is higher than that of the simulation result, which can be attributed to the influence of the amplifier circuit. To assess the device’s durability under a repeated stimulus, a typical 3200-cycle test was conducted at a constant hydrodynamic pressure gradient of 1127.4 Pa/m (Figure 12d,e). The results demonstrated that the DSHPS exhibited excellent durability and long-term stability, thus highlighting its potential for precise and prolonged monitoring of hydrodynamic stimuli.

In comparison to similar studies focusing on the development of artificial canal lateral lines or hydrodynamic pressure sensors, the DSHPS proposed in this study exhibits significant advantages in terms of pressure difference detection limitation and working water depth range (Figure 12f and Table S1 [15,16,25,27]). The exceptional hydrodynamic sensing performance of the DSHPS can be attributed to two key factors: (1) The utilization of a piezoelectric P(VDF-TrFE)/BTO nanofiber mat as the sensing material, which possesses excellent piezoelectric properties, enabling the detection of subtle water flow pressure signals. (2) The incorporation of an amplification circuit in the DSHPS effectively amplifies the piezoelectric signals from the sensing material, resulting in a high signal-to-noise ratio. The wide working water depth range achieved by the DSHPS is primarily attributed to (1) the implementation of a piezoelectric microcantilever-based sensing element and a bioinspired microchannel structure, and (2) the adoption of a high Young’s modulus packaging strategy inspired by deep-sea fish skulls, which protects the device from failure caused by the elevated hydrostatic pressure encountered at greater water depths.

### 3.4. Object Localization Performance of DSHPS

**Figure 13:**
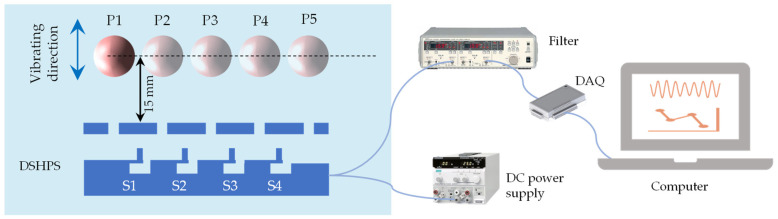
The experimental setup schematic for testing the dipole source localization response of the DSHPS.

The primary objective of the lateral line system is to aid fish in recognizing their surrounding environments, including object localization [45]. Object localization is also crucial in engineering applications, such as assisted navigation in unmanned underwater vehicles (UUVs). To assess the spatial location capability of the DSHPS, we conducted experiments using a dipole source. The established experimental setup is depicted in Figure 13. A sphere was vibrated perpendicular to the long axis of the DSHPS, positioned above the canal pores, while maintaining a constant vertical distance of 15 mm between the sphere and the DSHPS. The vibration frequency was maintained at 77 ± 3 Hz. The outputs of all four sensing elements within the DSHPS were simultaneously recorded and analyzed in response to five different dipole positions (P1, P2, P3, P4, and P5).

**Figure 14:**
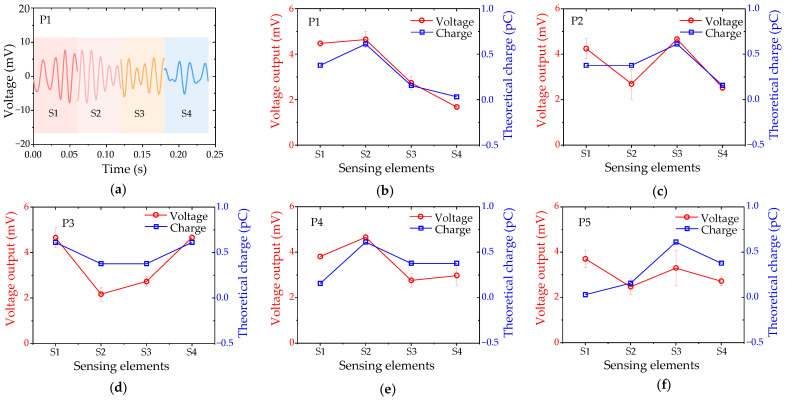
(**a**) Time-domain voltage outputs of the four sensing elements in the DSHPS when the dipole is positioned at position P1; (**b**–**f**) Measured voltage response curve and theoretically calculated charge response curve of the DSHPS in response to different dipole positions.

When the sphere was vibrated at position P1, the sensing elements demonstrated an excellent dynamic response to periodic hydrodynamic stimuli, as depicted in Figure 14a. Notably, sensing element 2 (S2) exhibited the highest voltage output, while sensing element 4 (S4) displayed the lowest voltage output. To quantitatively evaluate the amplitude response curve of the DSHPS, it was calculated accordingly. As expected, S2 yielded the highest voltage output, while S4 exhibited the lowest voltage output. Moreover, the measured voltage response curve of the DSHPS closely followed the trend observed in the theoretical charge response curve of the DSHPS, as shown in Figure 14b. The peak of the DSHPS response curve shifted in response to different positions of the vibrating sphere, as illustrated in Figure 14c–f. Even when the vertical distance between the dipole and the DSHPS increased to 37.5 mm, similar observations and conclusions were drawn (Figure S14). These findings indicate that the developed DSHPS holds significant potential for accurately determining the spatial location of a dipole.

**Figure 15:**
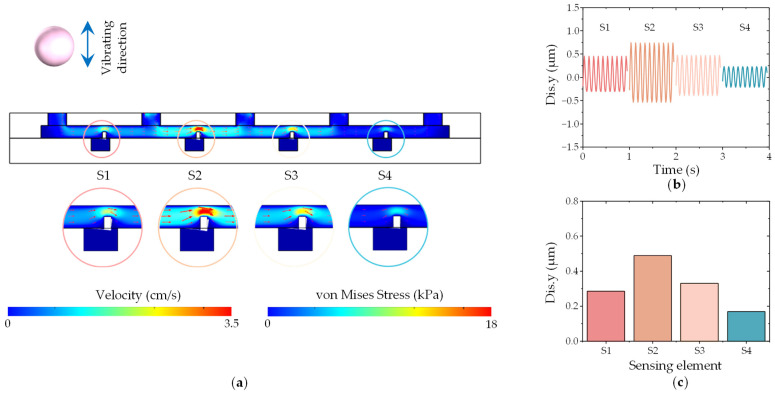
(**a**) Fluid–structure interaction (FSI) simulation illustrating the distribution of flow fields inside the microchannel and the deformation of the microcantilever; (**b**) Displacement of the cantilever beam corresponding to the four sensing elements (time series data) simulated using the finite element method (FEM) model; (**c**) Displacement amplitude response of the microcantilevers.

To gain a deeper understanding of the localization mechanism employed by the DSHPS, we conducted fluid–structure interaction (FSI) simulations to analyze the distribution of flow fields inside the microchannel and the displacement of the microcantilever. Considering computational efficiency, the vibration frequency of the sphere was set at 10 Hz. When the sphere vibrated at position P1, the flow field near sensing elements S2 and S4 exhibited the highest and lowest flow velocities, respectively, as depicted in Figure 15a and Movie S2. The interaction between the fluid and the microcantilever likely resulted in significant deformation in the microcantilever due to the high flow velocity. The displacement in the y-direction (Dis.y) of the microcantilever was extracted and analyzed. The results demonstrate that the microcantilever exhibits a dynamic response at a frequency of 10 Hz, matching the stimulus frequency. The displacement experienced by S2 was the highest, while S4 presented the lowest displacement (Figure 15b), mirroring the voltage outputs of the DSHPS shown in Figure 14a. Furthermore, the displacement amplitude of the microcantilever was evaluated. As anticipated, the displacement response curve of the microcantilever array aligned with the measured voltage response curve of the DSHPS (Figure 15c). Similar conclusions can be drawn when the dipole was located at other positions, such as P2 (Figure S15, Movie S3), P3 (Figure S16, Movie S4), P4 (Figure S17, Movie S5), and P5 (Figure S18, Movie S6), thereby validating the reliability of the experimental results obtained with the DSHPS.

### 3.5. Hydrodynamic Sensing Performance of DSHPS in Deep-Sea Environment

**Figure 16:**
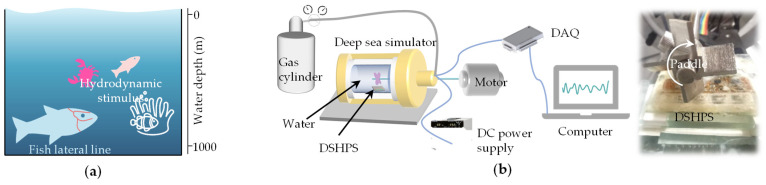
(**a**) Schematic illustrating typical hydrodynamic stimulation in deep-sea environments; (**b**) Schematic diagram and optical image of the experimental platform, simulating natural deep-sea conditions.

Deep-sea fish can use their own lateral line systems to perceive dynamic information in the environment (Figure 16a). To assess the hydrodynamic sensing capabilities of the DSHPS in deep-sea environments, we developed a deep-sea simulator (Figure 16b). This simulator consisted of three primary components: a high-pressure vessel filled with water, an excitation motor-driven paddle inserted into the vessel, and a nitrogen cylinder equipped with a pressure valve. By manipulating the nitrogen source, the pressure within the vessel could be regulated to replicate high static pressure conditions. The paddles were utilized to generate hydrodynamic disturbances, simulating the hydrodynamic stimuli encountered in deep-sea environments.

To assess the dynamic pressure generated by the paddles, we initially evaluated it using a commercial MS5401 pressure sensor. The time domain analysis indicated that the hydrodynamic pressure detected by the pressure sensor was approximately 15 Pa (Figure S19a). Furthermore, in terms of frequency response, the DSHPS demonstrated a dominant frequency of approximately 30 Hz, aligning with the disturbances generated by the paddles (Figure S19b). Given the limited space within the vessel, we focused on presenting two concise demonstrations to showcase the hydrodynamic sensing capabilities of the DSHPS in deep-sea environments. These demonstrations include the distance response of an individual sensor and the localization of hydrodynamic sources using a sensor array.

**Figure 17:**
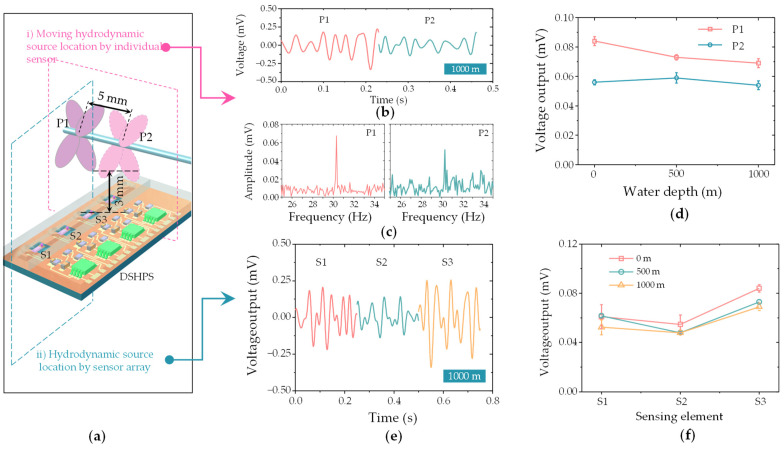
(**a**) Schematic representation of the DSHPS for detecting subtle hydrodynamic stimuli generated by moving objects; (**b**) Typical time-domain and (**c**) frequency-domain results of the DSHPS in a 1000-m water depth environment; (**d**) Amplitude response of the DSHPS to different stimulus positions under varying water depth conditions; (**e**) Typical time-domain voltage outputs of the three sensing elements in the DSHPS; (**f**) Amplitude response of the three sensing elements in the DSHPS to a constant stimulus position under different water depth conditions.

In the case of the distance response of an individual sensor, Position 1 (P1) refers to the paddles positioned directly above the canal pore, with the lower end of the blade located at a distance of 3 mm from the top surface of the DSHPS. Subsequently, Position 2 (P2) was situated 5 mm away from Position P1 along the rod’s direction (Figure 17a). Throughout the testing process, the paddles rotated at a constant speed of 450 ± 2 rpm, generating a hydrodynamic stimulus with a frequency of 30 Hz. The simulated water depths were set at 0 m, 500 m, and 1000 m, which were achieved by pressure on the vessel with values of 0 Pa, 5 MPa, and 10 MPa. The findings demonstrated that the DSHPS exhibited an excellent dynamic sensing performance, even in deep-sea hydrodynamic environments at a depth of 1000 m (Figure 17b,c). As clearly depicted in the frequency domain analysis, the DSHPS demonstrated a characteristic frequency of 30 Hz, which aligns consistently with the excitation frequency. The amplitude response analysis demonstrates that the voltage output of the sensing element associated with the paddles positioned at P2 was lower compared to that at P1, indicating a decrease in the pressure difference inside the canal as the paddles moved away from the DSHPS (Figure 17d). Importantly, this conclusion holds true irrespective of the water depth.

Finally, we assessed the localization performance of the DSHPS using three representative sensing elements. The paddles were positioned at P1, and the voltage outputs of the three sensing elements (S1, S2, S3) were simultaneously recorded. The DSHPS consistently displayed periodic voltage outputs (Figure 17e and Figure S20), aligning with the excitation frequency. The amplitude response analysis of the DSHPS revealed that S3 achieved the highest voltage outputs, likely attributed to experiencing the greatest pressure difference (Figure 17f). Importantly, this observation held true across different water depths. These results unequivocally validate the remarkable hydrodynamic sensing performance exhibited by the proposed DSHPS, even as the water depth increased from 0 m to 1000 m.

## 4. Conclusions

In this study, we have designed a DSHPS that utilizes P(VDF-TrFE)/BTO piezoelectric nanofibers for hydrodynamic flow field perception. Initially, a theoretical model was established to optimize the structure of the DSHPS. To enhance its signal transmission capabilities, an integrated pre-amplification circuit was designed. The amplification factor of this circuit was thoroughly investigated both theoretically and experimentally. The performance of the DSHPS was systematically evaluated under both shallow-water and deep-sea environments. The results demonstrate that the DSHPS achieves a pressure gradient detection limit of 14 Pa/m. Simulations and experimental research also reveal its dipole positioning capability. Notably, compared to existing CALL sensors, deep-sea hydrodynamic pressure detection demonstrates that the DSHPS has significant advantages in terms of subtle pressure variation detection even under 1000 m water depths. Therefore, the DSHPS offers significant potential for applications in high water depth conditions.

In future studies, our research aims to incorporate power supply and wireless transmission components into the circuitry to enable direct signal transmission. Additionally, we will explore the measurement of pressure in proximity to solid objects, akin to pressure measurements near underwater vehicles or submarines. In addition, the DSHPS has detected pressure variations under 1000 m water depths, and the result is in the absence of external disturbance. Later, we can try to add an external disturbance to detect the output performance of the DSHPS in the pressure chamber.

## Data Availability

The raw/processed data required to reproduce these findings cannot be shared at this time as the data also form part of an ongoing study.

## Supplementary Materials

The following supporting information can be downloaded at: https://www.mdpi.com/article/10.3390/biomimetics9030190/s1, Figure S1: XRD spectra of P(VDF-TrFE)/BTO nanofiber mat; Figure S2: FTIR spectra of P(VDF-TrFE)/BTO nanofiber mat; Figure S3: PFM amplitude and phase diagrams of P(VDF-TrFE)/BTO nanofiber; Figure S4: theoretical model creation of the cantilever; Figure S5: resonant frequency analysis of the cantilever; Figure S6: schematic illustrating the pre-amplification circuit; Figure S7: layout and developed pre-amplification circuit; Figure S8: magnification times of developed integrated pre-amplification circuits compared with theorical values; Figure S9: COMSOL model setup to simulate fluid–structure interactions of the DEHPS; Figure S10: stress distribution in the DSHPS with PDMS canal at 1000 m deep-sea environments; Figure S11: simulation results exhibits the shear stress experienced by electrical components within DSHPS packaged with PDMS; Figure S12: experimental platform validation using a commercial MS5401 pressure sensor; Figure S13: comparison of the voltage output between simulation and experimental results of proposed DSHPS device, responding to series of pressure differences in the shallow water environments; Figure S14: dipole source location at a vertical distance of 37.5 mm; Figures S15–S18: FSI analysis illustrating the location mechanism; Figure S19: hydrodynamic pressure detected by MS5401; Figure S20: FFT response of DSHPS responded to the subtle hydrodynamic stimulus generated by paddles, at a water depth of 1000 m; Table S1: performance comparison of flexible pressure sensors for hydrodynamic perception. Movie S1: Structure-electrostatic Multiphysics coupling simulations (Piezopotential distribution within piezoelectric film); Movie S2: FSI analysis for vibrating sphere location by DSHPS array (Position 1); Movie S3: FSI analysis for vibrating sphere location by DSHPS array (Position 2); Movie S4: FSI analysis for vibrating sphere location by DSHPS array (Position 3); Movie S5: FSI analysis for vibrating sphere location by DSHPS array (Position 4); Movie S6: FSI analysis for vibrating sphere location by DSHPS array (Position 5). References [46,47,48,49,50,51,52,53] are cited in the Supplementary Materials.

## References

[1] Skålvik A.M., Saetre C., Frøysa K.E., Bjørk R.N., Tengberg A. (2023). Challenges, Limitations, and Measurement Strategies to Ensure Data Quality in Deep-Sea Sensors. Front. Mar. Sci..

[2] Xie K., Li T., Zhang Y., Wu S., Yang C. (2023). Multiphysics Co-Simulation and Experimental Study of Deep-Sea Hydrothermal Energy Generation System. J. Mar. Sci. Eng..

[3] Mitra A., Panda J.P., Warrior H.V. (2019). The Effects of Free Stream Turbulence on the Hydrodynamic Characteristics of an AUV Hull Form. Ocean Eng..

[4] Liu K., Wang H., Xu X., Song T., Meng Q. (2022). Development and Trials of a Novel Deep-Sea Multi-Joint Autonomous Underwater Vehicle. Ocean Eng..

[5] Saponara S. (2018). Sensing and Connection Systems for Assisted and Autonomous Driving and Unmanned Vehicles. Sensors.

[6] Mitra A., Panda J.P., Warrior H.V. (2020). Experimental and Numerical Investigation of the Hydrodynamic Characteristics of Autonomous Underwater Vehicles over Sea-Beds with Complex Topography. Ocean Eng..

[7] Chen X., Zhou F., Li G., Cao X., Li T. (2022). Self-Powered Soft Robot in the Mariana Trench. Kexue Tongbao/Chin. Sci. Bull..

[8] Grelowska G., Kozaczka E. (2014). Underwater Acoustic Imaging of the Sea. Arch. Acoust..

[9] Rajapan D., Rajeshwari P.M., Zacharia S. Importance of Underwater Acoustic Imaging Technologies for Oceanographic Applications—A Brief Review. Proceedings of the OCEANS 2022—Chennai.

[10] Eren F., Pe’Eri S., Thein M.W., Rzhanov Y., Celikkol B., Swift M.R. (2017). Position, Orientation and Velocity Detection of Unmanned Underwater Vehicles (UUVs) Using an Optical Detector Array. Sensors.

[11] Kottapalli A.G.P., Asadnia M., Miao J., Triantafyllou M.S. (2016). Biomimetic Microsensors Inspired by Marine Life.

[12] Peleshanko S., Julian M.D., Ornatska M., McConney M.E., LeMieux M.C., Chen N., Tucker C., Yang Y., Liu C., Humphrey J.A.C. (2007). Hydrogel-Encapsulated Microfabricated Haircells Mimicking Fish Cupula Neuromast. Adv. Mater..

[13] Montgomery J.C., Baker C.F., Carton A.G. (1997). The Lateral Line can Mediate Rheotaxis in Fish. Nature.

[14] Kamat A.M., Zheng X., Bos J., Cao M., Triantafyllou M.S., Kottapalli A.G.P. (2023). Undulating Seal Whiskers Evolved Optimal Wavelength-to-Diameter Ratio for Efficient Reduction in Vortex-Induced Vibrations. Adv. Sci..

[15] Shaikh S.F., Mazo-Mantilla H.F., Qaiser N., Khan S.M., Nassar J.M., Geraldi N.R., Duarte C.M., Hussain M.M. (2019). Noninvasive Featherlight Wearable Compliant “Marine Skin”: Standalone Multisensory System for Deep-Sea Environmental Monitoring. Small.

[16] He Q., Zhang W., Sheng T., Gong Z., Dong Z., Zhang D., Jiang Y. (2022). Flexible Conductivity-Temperature-Depth-Strain (CTDS) Sensor Based on a CNT/PDMS Bottom Electrode for Underwater Sensing. Flex. Print. Electron..

[17] Jiang Y., Ma Z., Zhang D. (2019). Flow Field Perception Based on the Fish Lateral Line System. Bioinspir. Biomim..

[18] Zheng X., Kamat A.M., Krushynska A.O., Cao M., Kottapalli A.G.P. (2022). 3D Printed Graphene Piezoresistive Microelectromechanical System Sensors to Explain the Ultrasensitive Wake Tracking of Wavy Seal Whiskers. Adv. Funct. Mater..

[19] Liu G., Wang A., Wang X., Liu P. (2016). A Review of Artificial Lateral Line in Sensor Fabrication and Bionic Applications for Robot Fish. Appl. Bionics Biomech..

[20] Guo L., Xu K., Li J., Liu C. (2021). A MEMS Flow Sensor Based on Fish Lateral Line Sensing System. Microsyst. Technol..

[21] Han Z., Liu L., Wang K., Song H., Chen D., Wang Z., Niu S., Zhang J., Ren L. (2018). Artificial Hair-Like Sensors Inspired from Nature: A Review. J. Bionic Eng..

[22] Zhai Y., Zheng X., Xie G. (2021). Fish Lateral Line Inspired Flow Sensors and Flow-Aided Control: A Review. J. Bionic Eng..

[23] Asadnia M., Kottapalli A.G.P., Miao J., Warkiani M.E., Triantafyllou M.S. (2015). Artificial Fish Skin of Self-Powered Micro-Electromechanical Systems Hair Cells for Sensing Hydrodynamic Flow Phenomena. J. R. Soc. Interface.

[24] Sharif M.A.A., Tan X. A Pressure Gradient Sensor Inspired by the Canal Neuromasts of Fish. Proceedings of the Electroactive Polymer Actuators and Devices (EAPAD) XX.

[25] Jiang Y., Ma Z., Fu J., Zhang D. (2017). Development of a Flexible Artificial Lateral Line Canal System for Hydrodynamic Pressure Detection. Sensors.

[26] Gong L., Fu J., Ma Z., Zhang D., Jiang Y. Canal-Type Artificial Lateral Line Sensor Array Based on Highly Aligned P(VDF-TrFE) Nanofibers. Proceedings of the 2016 IEEE 11th Annual International Conference on Nano/Micro Engineered and Molecular Systems (NEMS).

[27] Ma Z., Xu Y., Jiang Y., Hu X., Zhang D. (2020). BTO/P(VDF-TrFE) Nanofiber-Based Artificial Lateral Line Sensor with Drag Enhancement Structures. J. Bionic Eng..

[28] Marshall N.J. (1996). The Lateral Line Systems of Three Deep-Sea Fish. J. Fish Biol..

[29] Lannoo M.J., Eastman J.T., Orr J.W. (2009). Nervous and Sensory Systems in Sub-Arctic and Antarctic Snailfishes of the Genus *Paraliparis* (Teleostei: Scorpaeniformes: Liparidae). Copeia.

[30] Reed A.J., Morris J.P., Linse K., Thatje S. (2013). Plasticity in Shell Morphology and Growth among Deep-Sea Protobranch Bivalves of the Genus *Yoldiella* (Yoldiidae) from Contrasting Southern Ocean Regions. Deep Sea Res. Part I Oceanogr. Res. Pap..

[31] Ying Z., Wang S., Wong W.C., Cai X., Feng X., Xiang S., Cai Y., Qian P.Y., Wang N. (2022). An Insight into the Microstructures and Composition of 2,700 m-Depth Deep-Sea Limpet Shells. Front. Mar. Sci..

[32] Hu X., Yan X., Gong L., Wang F., Xu Y., Feng L., Zhang D., Jiang Y. (2019). Improved Piezoelectric Sensing Performance of P(VDF-TrFE) Nanofibers by Utilizing BTO Nanoparticles and Penetrated Electrodes. ACS Appl. Mater. Interfaces.

[33] Shen Z., Lu J., Tan C.W., Miao J., Wang Z. (2013). D33 Mode Piezoelectric Diaphragm Based Acoustic Transducer with High Sensitivity. Sens. Actuators A Phys..

[34] Zhang X., Shan X., Xie T., Miao J. (2020). A New Sensor Inspired by the Lateral-Line System of Fish Using the Self-Powered D33 Mode Piezoelectric Diaphragm for Hydrodynamic Sensing. Mech. Syst. Signal Process..

[35] Windsor S.P., Norris S.E., Cameron S.M., Mallinson G.D., Montgomery J.C. (2010). The Flow Fields Involved in Hydrodynamic Imaging by Blind Mexican Cave Fish (*Astyanax fasciatus*). Part II: Gliding Parallel to a Wall. J. Exp. Biol..

[36] Valiantzas J.D. (2008). Explicit Power Formula for the Darcy–Weisbach Pipe Flow Equation: Application in Optimal Pipeline Design. J. Irrig. Drain. Eng..

[37] Khlapuk M., Bezusyak O., Volk L., Zhang Z. (2021). Theoretical Research of Friction Factor in Hydraulically Smooth Pipes. E3S Web Conf..

[38] Park K.I., Xu S., Liu Y., Hwang G.T., Kang S.J.L., Wang Z.L., Lee K.J. (2010). Piezoelectric BaTiO_3_ Thin Film Nanogenerator on Plastic Substrates. Nano Lett..

[39] Toprak A., Tigli O. (2014). Piezoelectric Energy Harvesting: State-of-the-Art and Challenges. Appl. Phys. Rev..

[40] Kim B.J., Meng E. (2016). Micromachining of Parylene C for BioMEMS. Polym. Adv. Technol..

[41] Wong E.H., Wong C.K. (2009). Approximate Solutions for the Stresses in the Solder Joints of a Printed Circuit Board Subjected to Mechanical Bending. Int. J. Mech. Sci..

[42] Liu C. (2007). Micromachined Biomimetic Artificial Haircell Sensors. Bioinspir. Biomim..

[43] McConney M.E., Chen N., Lu D., Hu H.A., Coombs S., Liu C., Tsukruk V.V. (2009). Biologically Inspired Design of Hydrogel-Capped Hair Sensors for Enhanced Underwater Flow Detection. Soft Matter.

[44] Yang Y., Klein A., Bleckmann H., Liu C. (2011). Artificial Lateral Line Canal for Hydrodynamic Detection. Appl. Phys. Lett..

[45] Goulet J., Engelmann J., Chagnaud B.P., Franosch J.M.P., Suttner M.D., Van Hemmen J.L. (2008). Object Localization through the Lateral Line System of Fish: Theory and Experiment. J. Comp. Physiol. A.

[46] Fu J., Jiang Y., Zhang D. PVDF Based Artificial Canal Lateral Line for Underwater Detection. Proceedings of the 2015 IEEE SENSORS.

[47] Ma Z., Jiang Y., Wu P., Xu Y., Hu X., Gong Z., Zhang D. (2019). Constriction Canal Assisted Artificial Lateral Line System for Enhanced Hydrodynamic Pressure Sensing. Bioinspiration Biomim..

[48] Fernandez V.I., Hou S.M., Hover F.S., Lang J.H., Triantafyllou M.S. (2007). Lateral-Line-Inspired Mems-Array Pressure Sensing for Passive Underwater Navigation.

[49] Herzog H., Klein A., Bleckmann H., Holik P., Schmitz S., Siebke G., Tätzner S., Lacher M., Steltenkamp S. (2015). μ-Biomimetic Flow-Sensors—Introducing Light-Guiding PDMS Structures into MEMS. Bioinspiration Biomim..

[50] Kottapalli A.G.P., Asadnia M., Miao J.M., Barbastathis G., Triantafyllou M.S. (2012). A Flexible Liquid Crystal Polymer MEMS Pressure Sensor Array for Fish-like Underwater Sensing. Smart Mater. Struct..

[51] Shi K., Sun B., Huang X., Jiang P. (2018). Synergistic Effect of Graphene Nanosheet and BaTiO_3_ Nanoparticles on Performance Enhancement of Electrospun PVDF Nanofiber Mat for Flexible Piezoelectric Nanogenerators. Nano Energy.

[52] Sengupta D., Kottapalli A.G.P., Chen S.H., Miao J.M., Kwok C.Y., Triantafyllou M.S., Warkiani M.E., Asadnia M. (2017). Characterization of Single Polyvinylidene Fluoride (PVDF) Nanofiber for Flow Sensing Applications. AIP Adv..

[53] Kalmijn A.J. (1988). Hydrodynamic and Acoustic Field Detection. Sensory Biology of Aquatic Animals.

