# Supplementary material for: A Highly Sensitive Deep-Sea Hydrodynamic Pressure Sensor Inspired by Fish Lateral Line
Source: Biomimetics (Basel). 2024 Mar 20;9(3):190. doi: 10.3390/biomimetics9030190 (PMC10968026; doi:10.3390/biomimetics9030190)
Supplement: Supplementary file 1 [file biomimetics-09-00190-s001.zip › Deep-ses hydrodynamic pressure sensor-ESM-biomimetics.pdf]

Supporting Information

A Highly Sensitive Deep-Sea Hydrodynamic Pressure Sensor Inspired by Fish Lateral Line

Xiaohe Hu [1], Zhiqiang Ma [2,3,*], Zheng Gong [2], Fuqun Zhao [1], Sheng Guo [1], Deyuan Zhang [2], and Yonggang Jiang [2,*]

[1] School of Mechanical, Electronic and Control Engineering, Beijing Jiaotong University, Beijing 100044, China
[2] School of Mechanical Engineering and Automation, Beihang University, Beijing 100191, China
[3] Department of Biomedical Engineering, City University of Hong Kong, 83 Tat Chee Avenue, Kowloon, Hong Kong 999077, China
* Correspondence: zqma@hkcoche.org (Z.M.); jiangyg@buaa.edu.cn (Y.J.)

Supplementary Notes

Note S1. Piezoelectric phase evaluation of P(VDF-TrFE)/BTO nanofiber

The piezoelectricity of P(VDF-TrFE)/BTO nanofiber mat is determined by the crystallinity. There are mainly three crystal phases in the P(VDF-TrFE) molecular chain, namely α, β and γ phases. Among them, the β phase has piezoelectricity and plays a decisive role in the piezoelectricity of P(VDF-TrFE)/BTO nanofiber mat. The crystallinity of the β phase can be analyzed by X-ray diffraction (XRD) and Fourier Transform Infrared Spectroscopy (FTIR).

The BTO was used as the doping material for P(VDF-TrFE)/BTO nanofiber mat. In order to achieve the best piezoelectric performance of the P(VDF-TrFE)/BTO nanofiber mat, we optimized the BTO content, which can be referred to in our previous papers [1]. When the BTO content is 5 wt%, the crystallinity of the β phase is better than that of the nanofiber mat with doping ratios of 0 wt %, 9 wt% and 13 wt%.

The molecules in organic matter can obtain energy by absorbing certain wavelengths of infrared light, causing the chemical bonds or functional groups in the molecules to vibrate or rotate, resulting in the transition of molecular energy levels. The vibration of chemical bonds is mainly divided into stretching vibration and bending vibration. The chemical bonds or groups that make up the molecule correspond to specific infrared absorption peaks. The structure of the corresponding chemical bond or functional group can be determined by detecting the absorption of infrared light.

P(VDF-TrFE) copolymer is a chain structure formed by the head-to-tail connection of $-CF_2CH_2-$ and $-CF_2CFH-$ molecular chains. In order to further demonstrate the effect of BTO on the crystallinity of piezoelectric fibers, FTIR spectroscopy was used to characterize the crystallinity of P(VDF-TrFE)/BTO fibers. The FTIR result is shown in Figure S3. It is known that the absorption peaks at 846 and 1288 cm^{-1} are related to the symmetric stretching vibration of the $-CF_2$ functional group, and the absorption peak at 1400 cm^{-1} reflects the wagging vibration of $-CH_2$ in the β molecular chain. The related band at 765 cm^{-1} reflects the $-CF_2$ bending vibration of the α phase. According to the Beer-Lambert law [2,3], the content of the β phase can be calculated by the following formula:

$$F(\beta)=\frac{A_\beta}{1.26A_\alpha+A_\beta} \qquad (S11)$$

wherein, α and β correspond to the absorption spectra at 765 cm^{-1} and 846 cm^{-1}, respectively. When the BTO content is 5 wt%, the crystallinity of β phase in P(VDF-TrFE)/BTO nanofiber mat reaches 81%, which is better than other doped BTO contents.

Note S2. Electrode configuration optimization

The piezoelectric constant of the P(VDF-TrFE)/BTO nanofiber mat is related to the electrode structure. There are two working modes of P(VDF-TrFE)/BTO nanofiber mat, d_{31} and d_{33} modes. The d_{33} working mode of the nanofiber mat can be achieved by combining interdigital electrodes with cantilever structures. During the deformation of the piezoelectric cantilever, the internal stress is along the length of the cantilever (direction 1), and the polarization directions of the upper and lower electrodes are perpendicular to the length of the cantilever (direction 3). That is, the stress direction and the polarization direction are perpendicular to each other, and the piezoelectric constant is d_{31} mode. The interdigital electrodes make the polarization direction of the piezoelectric cantilever consistent with the stress direction, which is the d_{33} mode. For interdigital electrodes, the output voltage of the piezoelectric nanofiber mat is determined by the spacing of the interdigital electrodes. Compared to the d_{33} mode, the d_{31} mode is not efficient in piezoelectric sensors with the same structure and stress conditions. The reason is that the d_{33} parameter is typically twice the value of the d_{31} parameter [4,5]. In addition, interdigital electrodes are located below the piezoelectric nanofiber mat, which helps to protect the electrode layer from signal interference. The interdigital electrodes can be distributed on the same plane, which reduces the processing complexity. The advantages of interdigital electrodes as summarized as: 1) Convert piezoelectric mode to improve the piezoelectric output of the sensor. 2) Avoid signal interference and improve measurement accuracy. 3) Simplifying the processing technology.

Note S3. Theoretical calculation of DSHPS

Under the action of an external flow, pressure difference between adjacent canal pores is generated and can be expressed as $\triangle P$=$P1$-$P2$. The fluid inside the microfluidic canal is driven by the pressure difference and moves at a speed of v. Fluid motion exerts drag force on the cilium attached on the apex of the cantilever and bends the cantilever with a moment of M. The P(VDF-TrFE)/BTO nanofiber mat dispersed in the cantilever deflects and generates charges.

During the theoretical modeling of the DSHPS, it is believed that the fluid in the canal is considered to be viscous and non-compressed fluid. The fluid moves inside the canal, causing energy loss and pressure drop due to the frictional resistance of the canal walls. Based on Bernoulli equation and Darcy-Weisbach formula [6,7], the flow field relationship inside the canal is given by:

$$\frac{P_1}{\rho g}+\frac{v^2}{2g}=\frac{P_2}{\rho g}+\frac{v^2}{2g}+H_{\mathrm{f}} \tag{S1}$$

where v is the average flow velocity inside the canal, ρ is the fluid density, g is the acceleration of gravity. H_{f} is the head loss along the canal due to the frictional resistance, which can be expressed as [8]:

$$H_{\mathrm{f}}=\lambda\frac{1}{4R}\frac{v^2}{2g} \tag{S2}$$

where l is the spacing between adjacent canal pores, R is the hydraulic radius, and λ is the coefficient of frictional resistance, which is related to the Reynolds number (R_{e}) and the roughness of the canal, λ is defined by:

$$\lambda=\frac{64}{R_{\mathrm{e}}}=\frac{16\mu}{\rho vR} \tag{S3}$$

where μ is the fluid dynamic viscosity. The average velocity of fluid in the canal can be written as:

$$v=\frac{\Delta PR^2}{2\mu l} \tag{S4}$$

The viscous resistance F received by the cilium and the mechanical bending moment M of the cantilever can be calculated by the following equations:

$$F=\frac{1}{2}\rho v^2C_{\mathrm{d}}S \tag{S5}$$

$$M=F\frac{H}{2} \tag{S6}$$

where C_{d} is the dimensionless pressure coefficient, S is the cross-sectional area of the cilium and can be expressed as S=DH, D and H are the diameter and height of the cilium.

The Parylene C layer on the upper layer of the P(VDF-TrFE)/BTO nanofiber mat is relatively thin and is neglected in the design process. According to Hooke's law, the bending stress generated in the P(VDF-TrFE)/BTO nanofiber mat can be calculated as:

$$\sigma_1=E_1\varepsilon_1=\frac{E_1z}{\rho} \tag{S7}$$

$$\sigma_2=E_2\varepsilon_2=\frac{E_2 z}{\rho} \tag{S8}$$

where E_1 and E_2 are the Young's modulus of the P(VDF-TrFE)/BTO nanofiber mat and Parylene C, I_1 and I_2 are the moments of inertia around the neutral axis for the P(VDF-TrFE)/BTO nanofiber mat and Parylene C, c is the distance from the upper surface of the cantilever beam to the neutral layer, t_1 is the thickness of the P(VDF-TrFE)/BTO nanofiber mat.

The generated charge Q can be expressed as follows:

$$Q=d_{33}\sigma_1 A=\frac{21 d_{33} E_1 t_1 (E_2 t_2^2 - E_1 t_1^2)\rho C_d D H^2 \Delta P^2 R^4}{4\mu^2 l^2 (E_1 t_1^3 + E_2 t_2^3)(E_1 t_1 + E_2 t_2)} \tag{S9}$$

where d_{33} is the piezoelectric charge coefficient of the P(VDF-TrFE)/BTO nanofiber mat, t_2 is the thickness of the Parylene C substrate. Therefore, Q can be simplified as the following formula：

$$Q \propto t_1^{-n} D H^2 \Delta P^2 \tag{S10}$$

where n is a positive value. As can be seen that Q is proportional to the thickness of the P(VDF-TrFE)/BTO nanofiber mat, diameter and height of the cilium.

Note S4. Dipole source platform calibration

In the dipole source platform calibration process, the dipole vibrates perpendicular to the sensor. The pressure generates by the dipole can be calculated using the following formula [9]:

$$P(l)=\frac{2\pi^2 f^2 \rho s a^3}{l^2} \tag{S12}$$

where ρ is the density of water, f and s are the vibration frequency and amplitude of the dipole, l is the distance between the dipole center and the sensor.

The MS5401 with a sensitivity of 1.15 mV/Pa was employed to calibrate the dipole source platform. The dipole vibrated perpendicularly to the MS5401 at a frequency of 80±3 Hz. The pressure generated by the dipole was adjusted by changing the distance between the center of the dipole and the MS5401. During the test, a DC regulated power supply was used to provide ±2.5 V voltage. The output signal was filtered by a filter and then collected by a data acquisition card.

References

1. Hu, X.; Yan, X.; Gong, L.; Wang, F.; Xu, Y.; Feng, L.; Zhang, D.; Jiang, Y. Improved Piezoelectric Sensing Performance of P(VDF-TrFE) Nanofibers by Utilizing BTO Nanoparticles and Penetrated Electrodes. *ACS Appl. Mater. Interfaces* **2019**, *11*, 7379–7386.
2. Shi, K.; Sun, B.; Huang, X.; Jiang, P. Synergistic Effect of Graphene Nanosheet and BaTiO3 Nanoparticles on Performance Enhancement of Electrospun PVDF Nanofiber Mat for Flexible Piezoelectric Nanogenerators. *Nano Energy* **2018**, *52*, 153–162.
3. Sengupta, D.; Kottapalli, A.G.P.; Chen, S.H.; Miao, J.M.; Kwok, C.Y.; Triantafyllou, M.S.; Warkiani, M.E.; Asadnia, M. Characterization of Single Polyvinylidene Fluoride (PVDF) Nanofiber for Flow Sensing Applications. *AIP Adv.* **2017**, *7*.
4. Shen, Z.; Lu, J.; Tan, C.W.; Miao, J.; Wang, Z. D33 Mode Piezoelectric Diaphragm Based Acoustic Transducer with High Sensitivity. *Sensors Actuators, A Phys.* **2013**, *189*, 93–99.
5. Zhang, X.; Shan, X.; Xie, T.; Miao, J. A New Sensor Inspired by the Lateral-Line System of Fish Using the Self-Powered D_{33} Mode Piezoelectric Diaphragm for Hydrodynamic Sensing. *Mech. Syst. Signal Process.* **2020**, *141*, 106476.
6. Windsor, S.P.; Norris, S.E.; Cameron, S.M.; Mallinson, G.D.; Montgomery, J.C. The Flow Fields Involved in Hydrodynamic Imaging by Blind Mexican Cave Fish (Astyanax Fasciatus). Part II: Gliding Parallel to a Wall. *J. Exp. Biol.* **2010**, *213*, 3832–3842.
7. Valiantzas, J.D. Explicit Power Formula for the Darcy–Weisbach Pipe Flow Equation: Application in Optimal Pipeline Design. *J. Irrig. Drain. Eng.* **2008**, *134*, 454–461.
8. Khlapuk, M.; Bezusyak, O.; Volk, L.; Zhang, Z. Theoretical Research of Friction Factor in Hydraulically Smooth Pipes. *E3S Web Conf.* **2021**, *280*, 1–7.
9. Kalmijn, A.J. Hydrodynamic and Acoustic Field Detection. *Sens. Biol. Aquat. Anim.* **1988**, 83–130.

Supplementary Figures

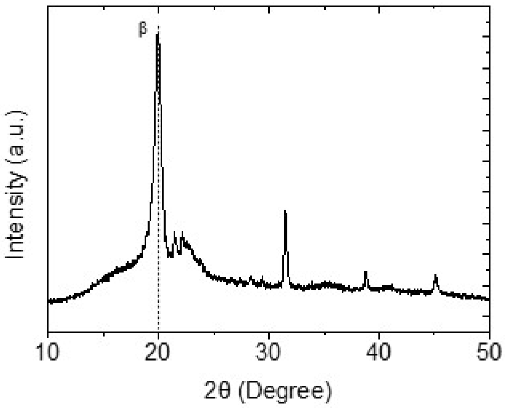

Figure S1. XRD spectra of P(VDF-TrFE)/BTO nanofiber mat.

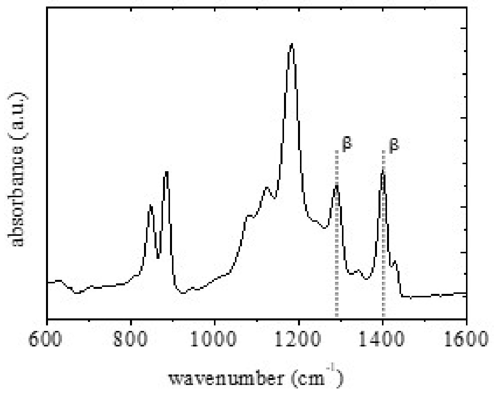

Figure S2. FTIR spectra of P(VDF-TrFE)/BTO nanofiber mat.

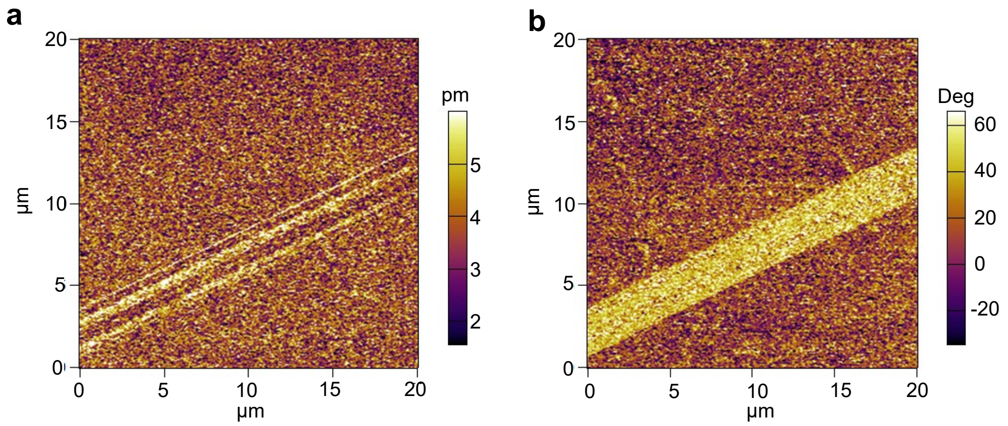

Figure S3. PFM amplitude and phase diagrams of P(VDF-TrFE)/BTO nanofiber.

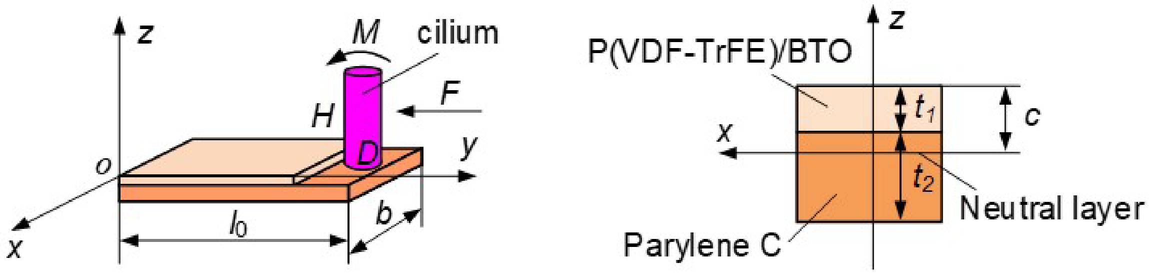

Figure S4. Theoretical model creation of the cantilever.

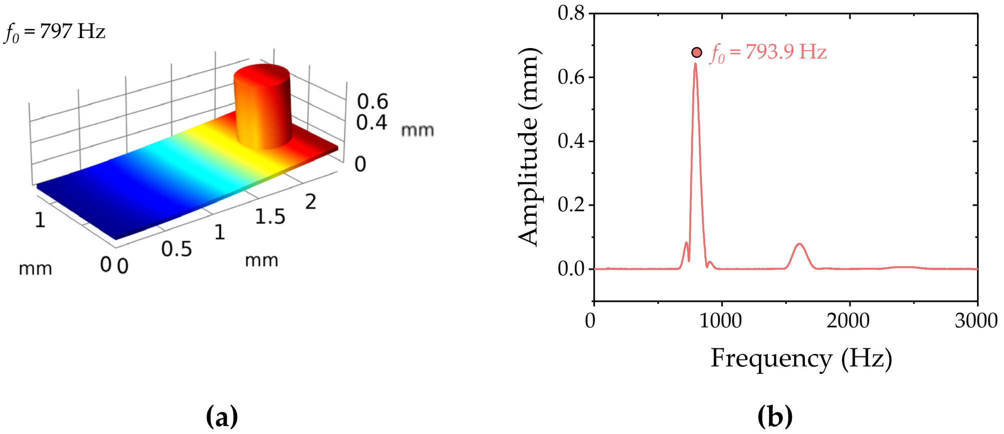

Figure S5. Resonant frequency analysis of the cantilever. (**a**) 3D simulation result; (**b**) Evaluated by Laser Doppler Velocimetry (LDV).

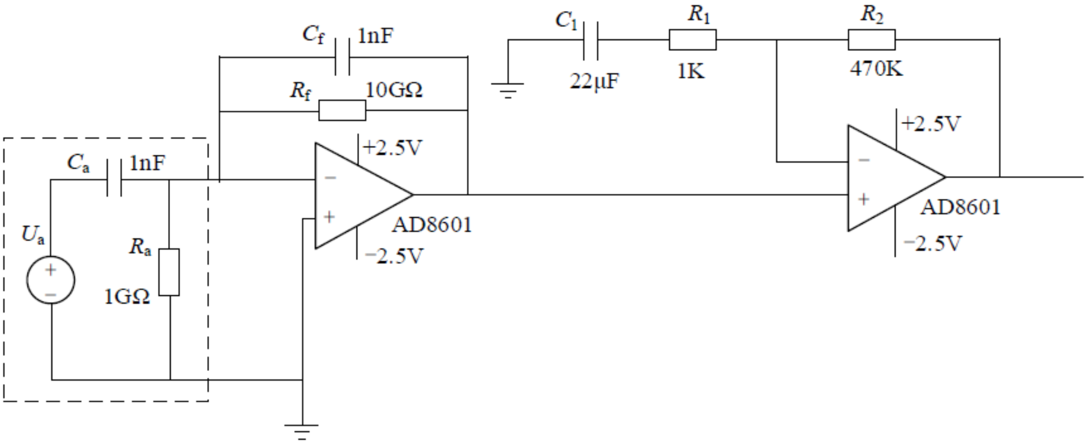

Figure S6. Schematic illustrating the pre-amplification circuit.

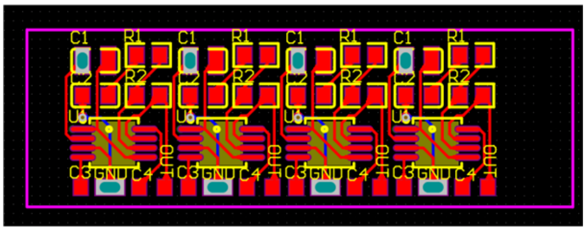

(a)

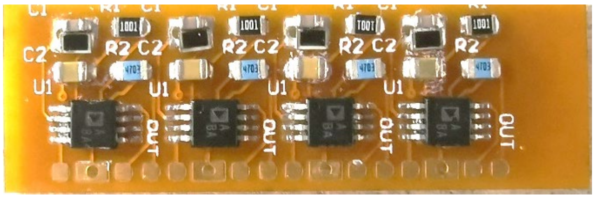

(b)

Figure S7. The layout and developed pre-amplification circuit. (**a**) Schematic diagram of the printed wire layout on the PCB; (**b**) The developed pre-amplification circuit.

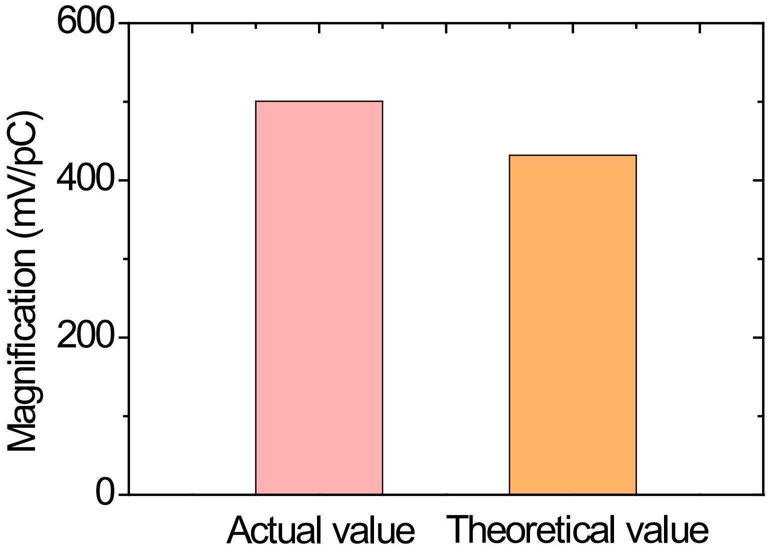

Figure S8. Magnification times of developed integrated pre-amplification circuits compared with theorical values.

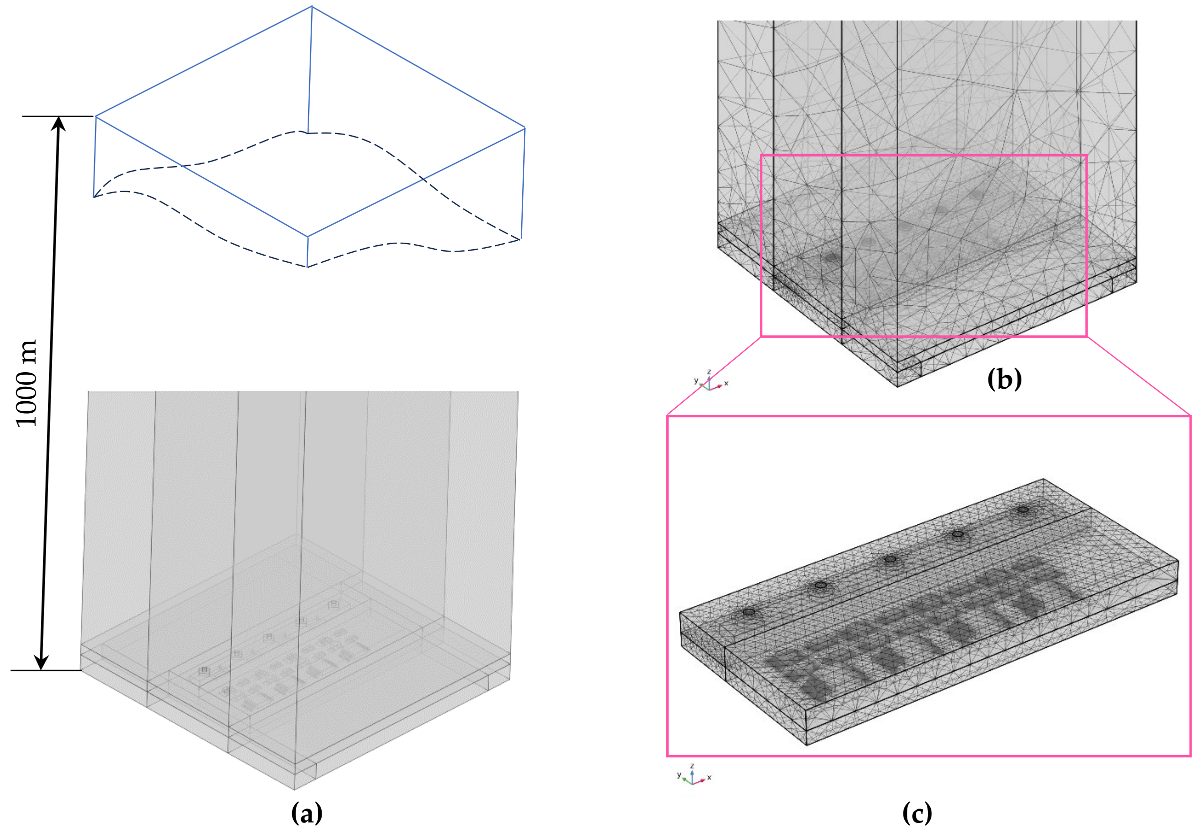

Figure S9. COMSOL model setup to simulate fluid-structure interactions of the DEHPS. (**a**) Overall structure diagram of the FEA configuration under 1000 m hydrodynamic environment; (**b**, **c**) Mesh generation of the DSHPS.

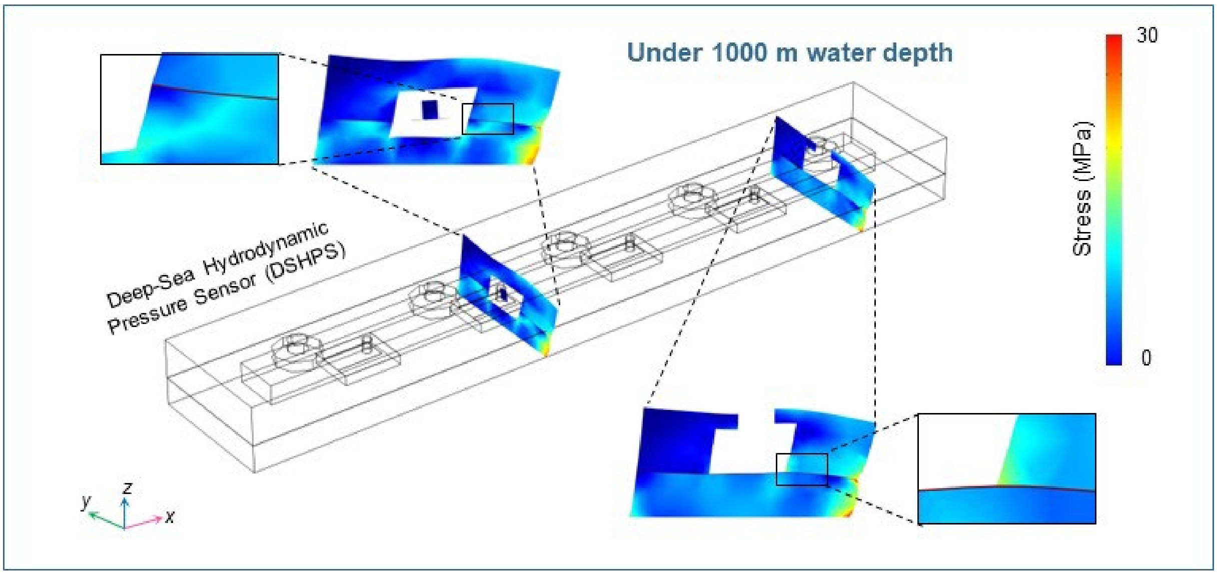

Figure S10. Stress distribution in the DSHPS with PDMS canal at 1000 m deep-sea environments.

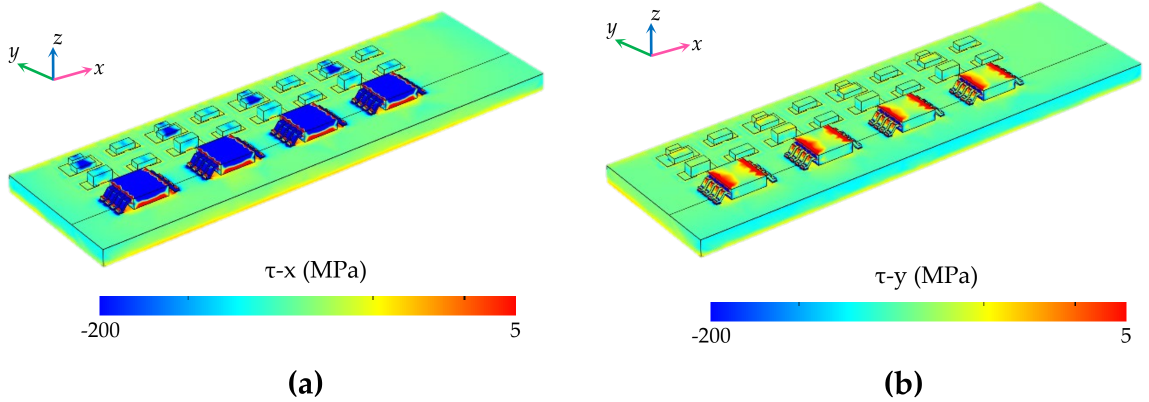

Figure S11. Simulation results exhibits the shear stress experienced by electrical components within DSHPS packaged with PDMS. (**a**) Distribution of shear stress in the *x*-direction within the DSHPS; (**b**) Distribution of shear stress in the *y*-direction within the DSHPS.

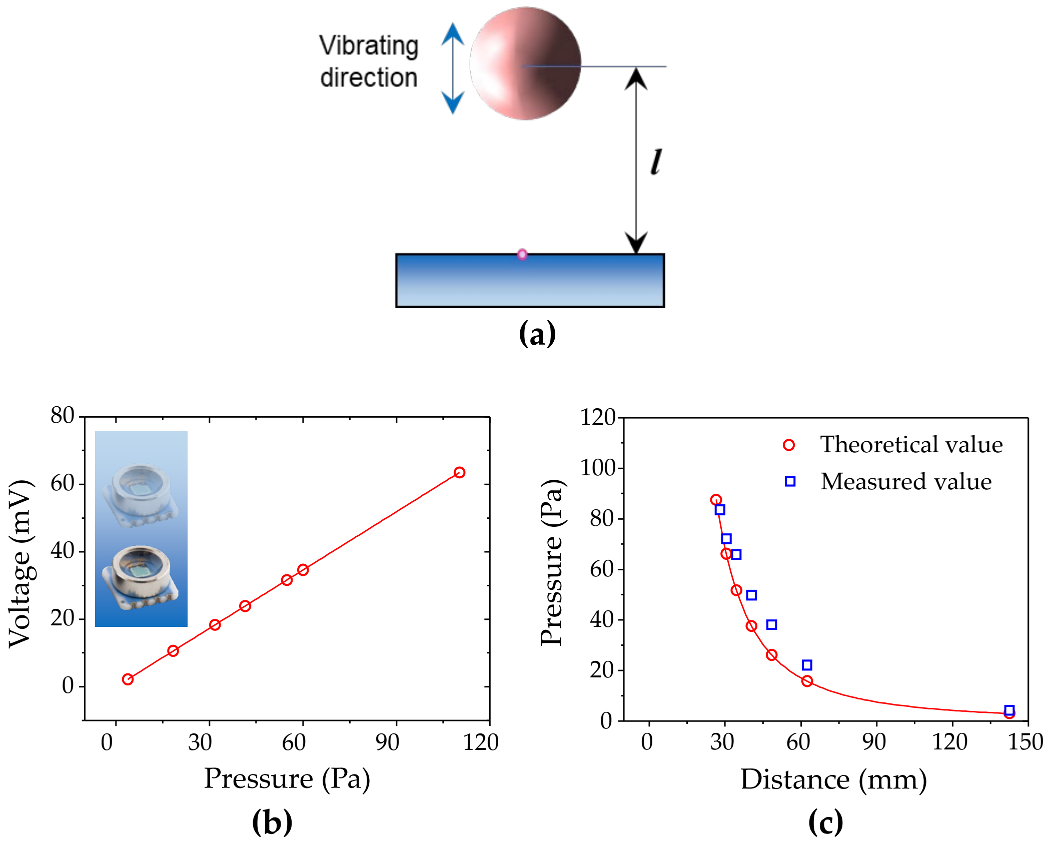

Figure S12. Experimental platform validation using a commercial MS5401 pressure sensor. (**a**) Schematic illustrating the hydrodynamic pressure validation platform; (**b**) Hydrostatic pressure response curve, the pressure sensitivity was calculated as 1.15 mV/Pa; (**c**) Pressure response of the MS5401 to different dynamic stimuli generated by dipole source.

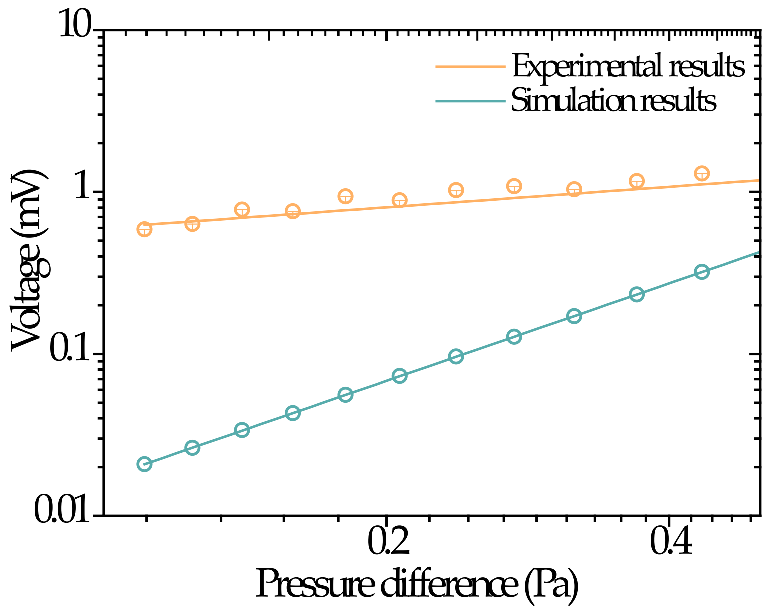

Figure S13. Comparison of the voltage output between simulation and experimental results of proposed DSHPS device, responding to series of pressure differences in the shallow water environments.

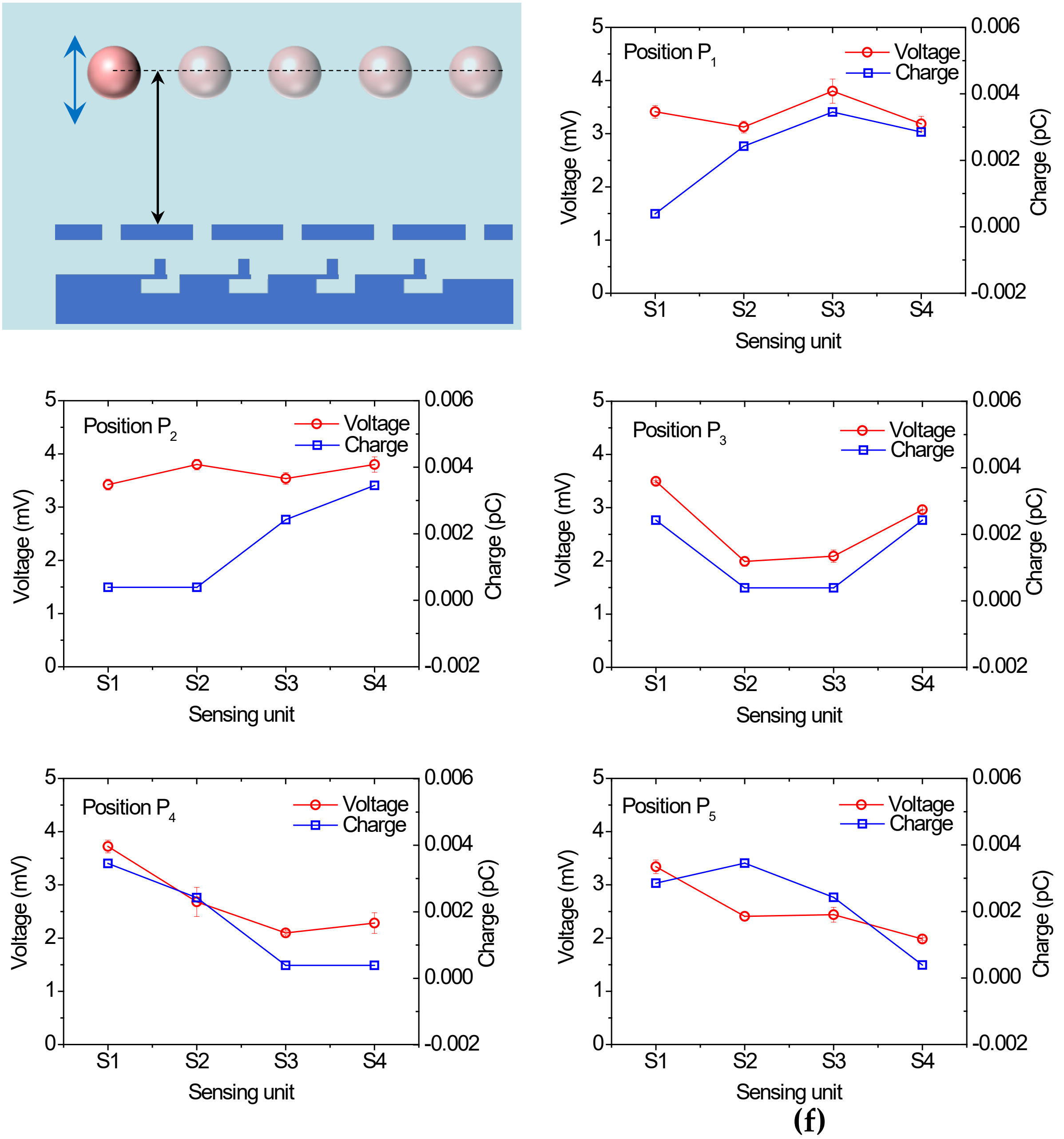

Figure S14. Dipole source location at a vertical distance of 37.5 mm. (a) Schematic diagram of testing mechanism; (b-f) The relationship between output voltage of the sensing units and the theoretical charge when the dipole was located at the five different positions.

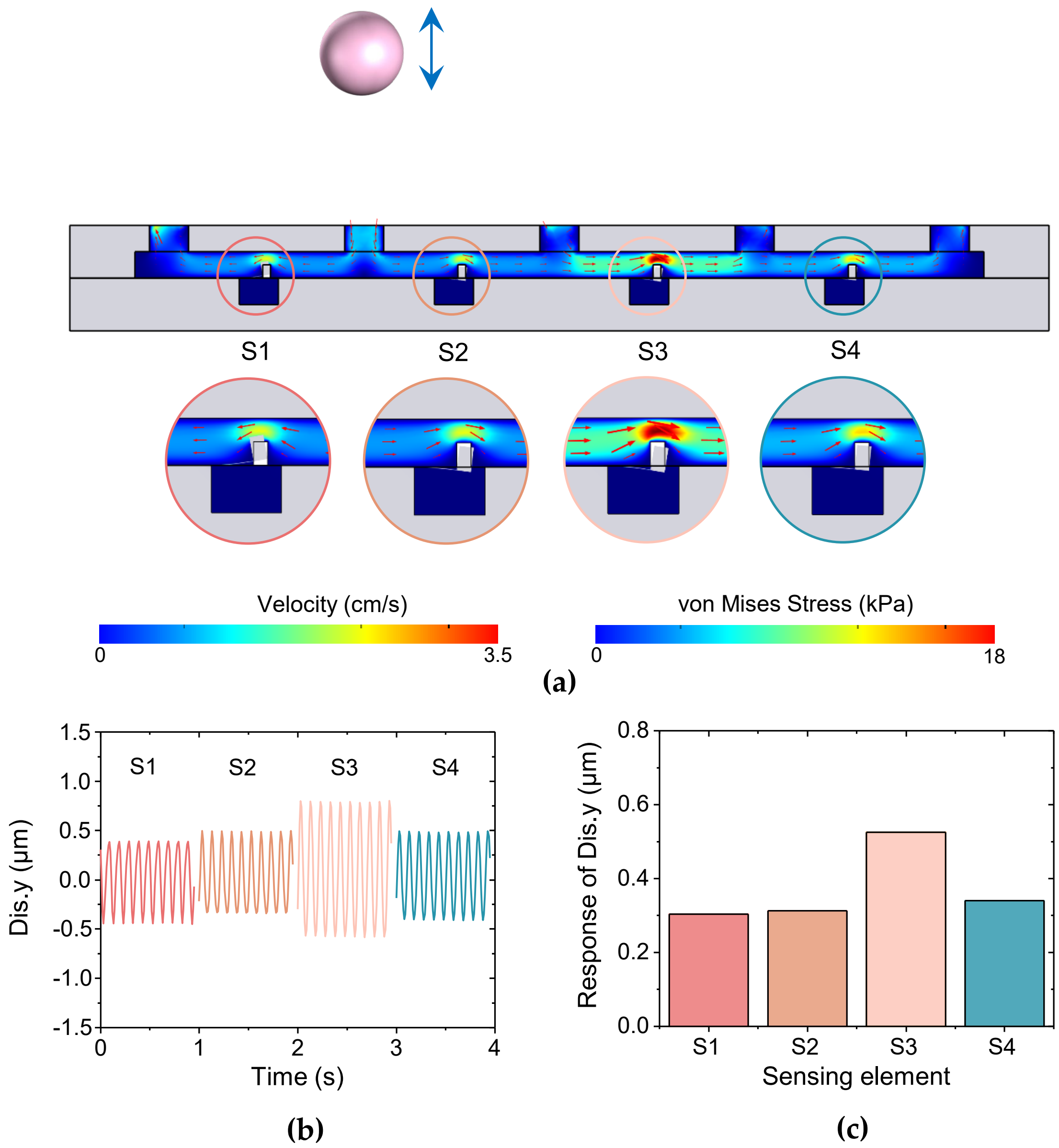

Figure S15. FSI analysis illustrating the location mechanism. Vertical distance was maintained as 15 mm. The dipole source was located at P2. (**a**) Simulation results of the velocity profile in the canal; (**b**) The displacement of the cantilever beam ends corresponding to the four sensing elements in time domain; (**c**) Displacement distribution results.

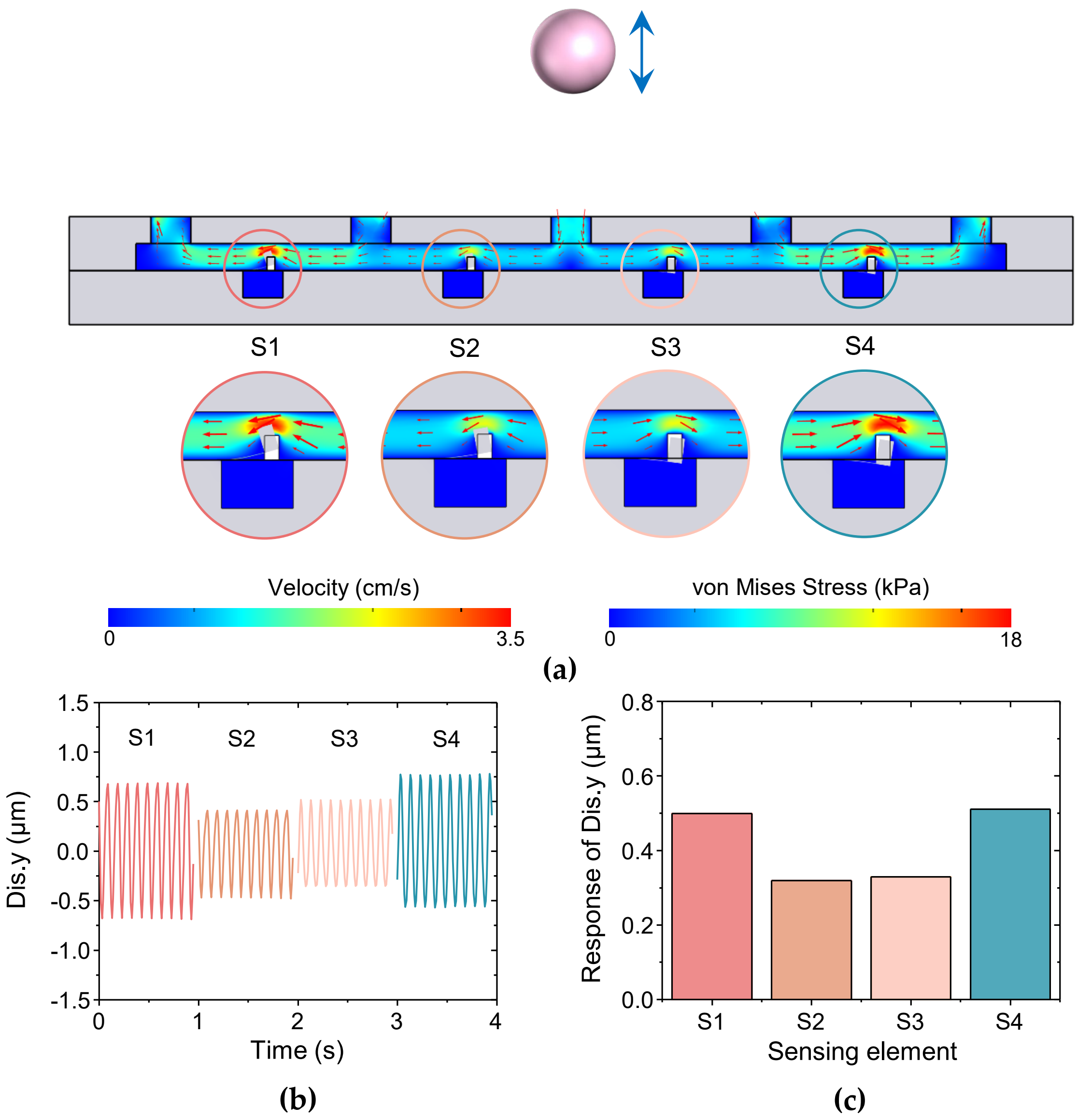

Figure S16. FSI analysis illustrating the location mechanism. Vertical distance was maintained as 15 mm. The dipole source was located at P3. (a) Simulation results of the velocity profile in the canal; (b) The displacement of the cantilever beam ends corresponding to the four sensing elements in time domain; (c) Displacement distribution results.

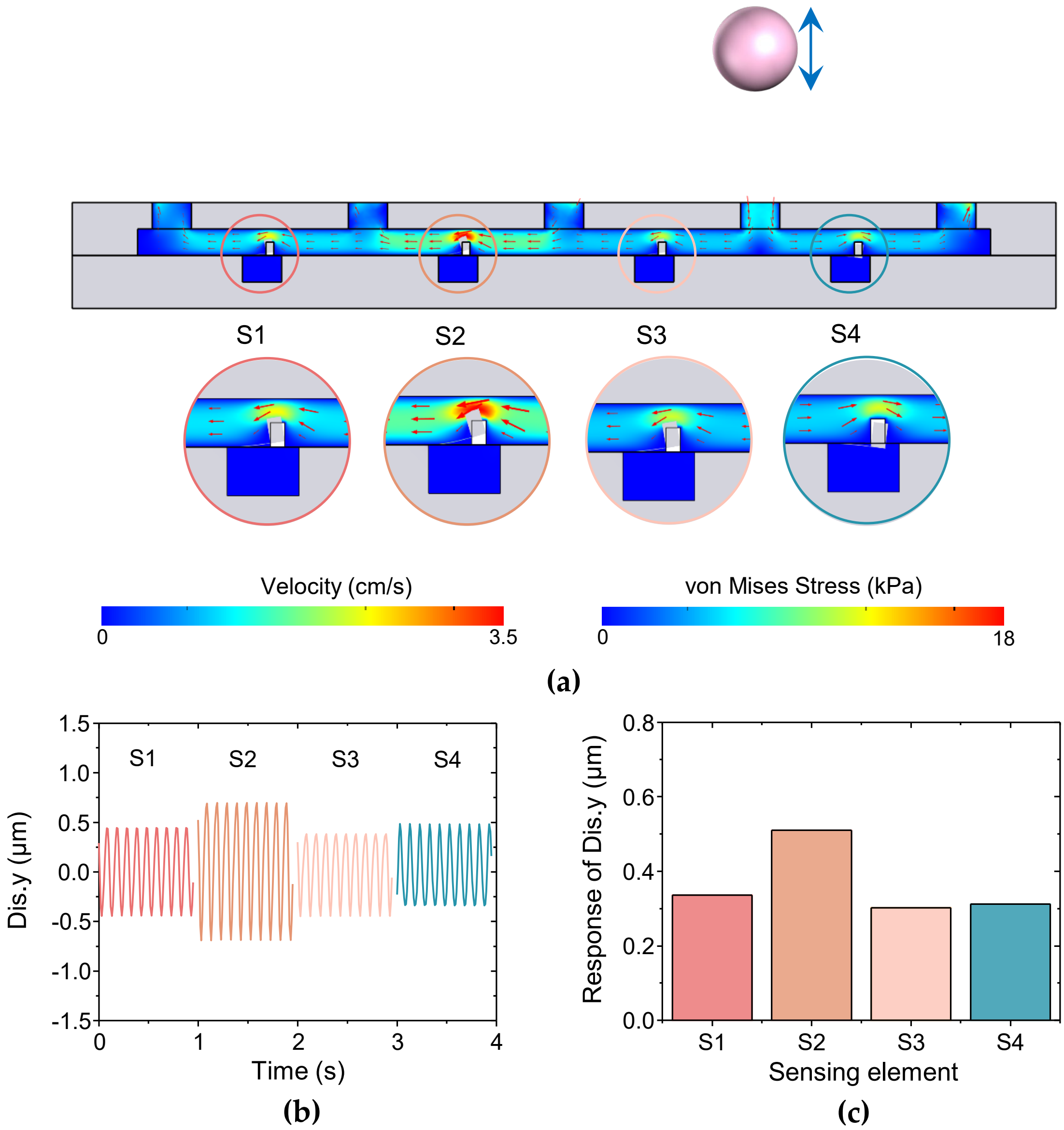

Figure S17. FSI analysis illustrating the location mechanism. Vertical distance was maintained as 15 mm. The dipole source was located at P4. (a) Simulation results of the velocity profile in the canal; (b) The displacement of the cantilever beam ends corresponding to the four sensing elements in time domain; (c) Displacement distribution results.

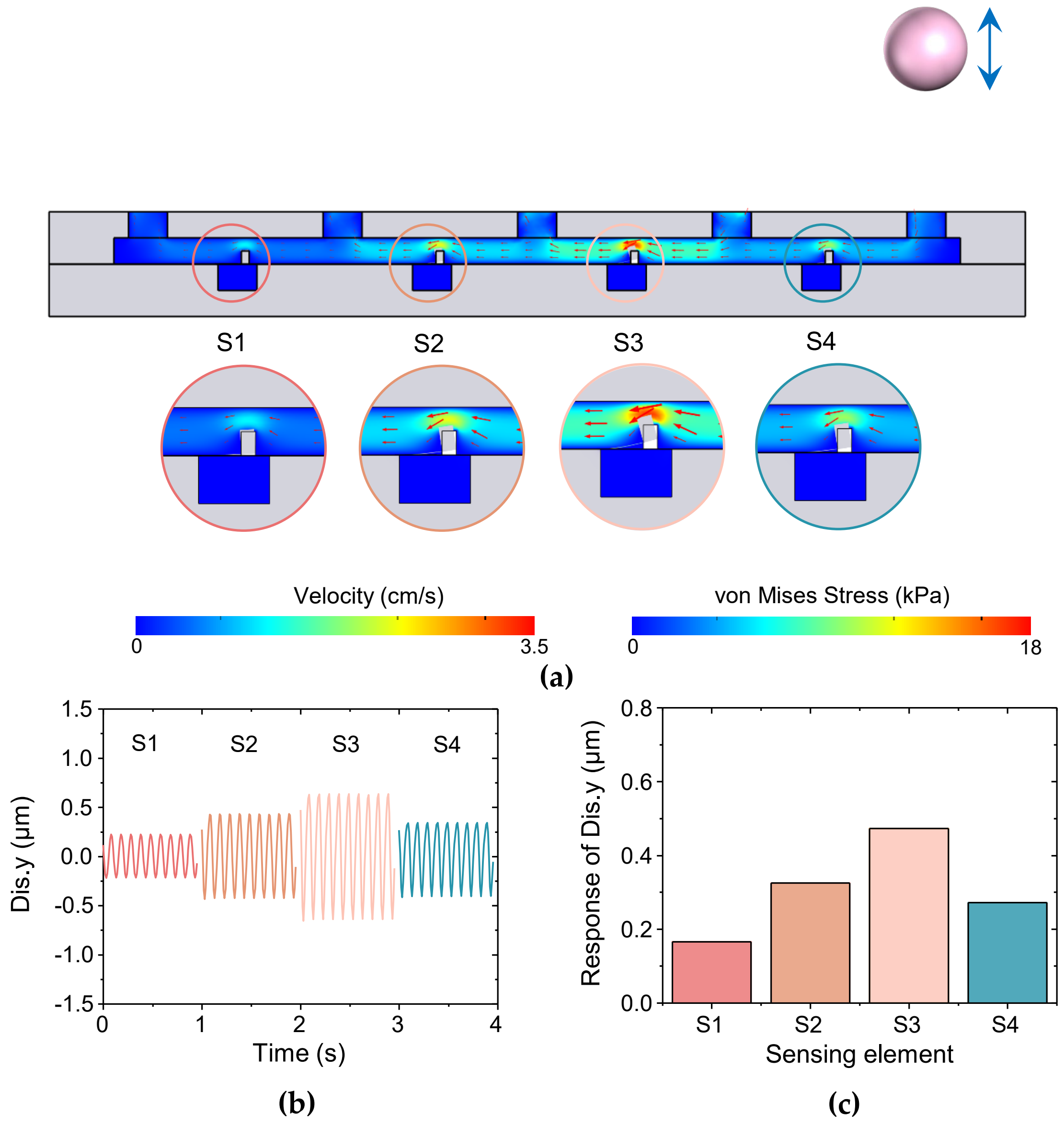

Figure S18. FSI analysis illustrating the location mechanism. Vertical distance was maintained as 15 mm. The dipole source was located at P5. (a) Simulation results of the velocity profile in the canal; (b) The displacement of the cantilever beam ends corresponding to the four sensing elements in time domain; (c) Displacement distribution results.

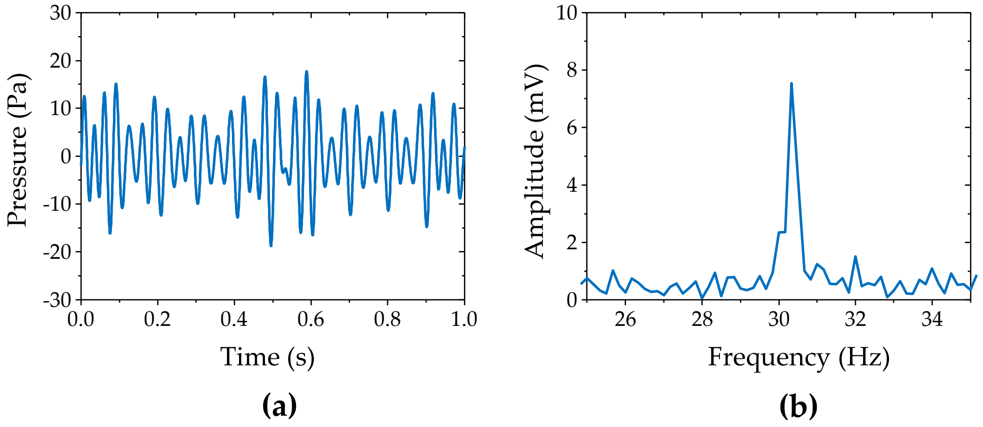

Figure S19. Hydrodynamic pressure detected by MS5401. (a) Commercial MS5401 response to the subtle hydrodynamic stimulus generated by paddles. It was evident that the hydrodynamic pressure was as low as approximately 15 Pa. (b) FFT result of the MS5401 to the paddles.

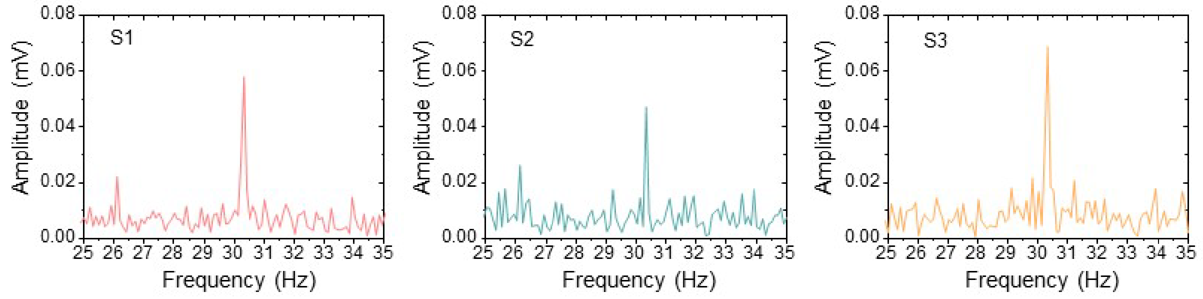

Figure S20. (a-c) FFT response of DSHPS responded to the subtle hydrodynamic stimulus generated by paddles, at a water depth of 1000 m.

Supplementary Table

Table S1. Performance comparison of flexible pressure sensors for hydrodynamic perception.

Sensing Material	Dynamic pressure detection limit	Water depth	High pressure resistance	Dynamic pressure sensing	Ref.
PVDF sheet	0.11 Pa	0.3 m	No	YES	Jiang *et al* [R1]
PVDF	3.1 Pa	0.3 m	No	YES	Fu *et al* [R2]
PVDF	0.0032 Pa	0.3 m	No	YES	Ma *et al* [R3]
P(VDF-TrFE)/BTO Nanofiber mat	0.07 Pa	0.4 m	No	YES	Ma *et al* [R4]
silicon diaphragm	1 Pa	shallow water	No	YES	Fernandez *et al* [R5]
light-guided PDMS lamella	<2.6 Pa	shallow water	No	YES	Herzog *et al* [R6]
gold piezoresistors	>75 Pa	0.4 m	No	YES	Kottapalli *et al* [R7]
dielectric PDMS	N/A	2000 m	YES	No	Shaikh *et al* [R8]
dielectric PDMS	N/A	500 m	YES	No	He *et al* [R9]
P(VDF-TrFE)/BTO Nanofiber mat	0.105 Pa	1000 m	YES	YES	This work

References:

R1. Jiang, Y.; Ma, Z.; Fu, J.; Zhang, D. Development of a Flexible Artificial Lateral Line Canal System for Hydrodynamic Pressure Detection. *Sensors (Switzerland)* **2017**, *17*.

R2. Fu, J.; Jiang, Y.; Zhang, D. PVDF Based Artificial Canal Lateral Line for Underwater Detection. *2015 IEEE SENSORS - Proc.* **2015**, 1–4.

R3. Ma, Z.; Jiang, Y.; Wu, P.; Xu, Y.; Hu, X.; Gong, Z.; Zhang, D. Constriction Canal Assisted Artificial Lateral Line System for Enhanced Hydrodynamic Pressure Sensing. *Bioinspiration and Biomimetics* **2019**, *14*.

R4. Ma, Z.; Xu, Y.; Jiang, Y.; Hu, X.; Zhang, D. BTO/P(VDF-TrFE) Nanofiber-Based Artificial Lateral Line Sensor with Drag Enhancement Structures. *J. Bionic Eng.* **2020**, *17*, 64–75.

R5. Fernandez, V.I.; Hou, S.M.; Hover, F.S.; Lang, J.H.; Triantafyllou, M.S. Lateral-Line-Inspired Mems-Array Pressure Sensing for Passive Underwater Navigation. *MIT Sea Grant Progr.* **2007**, 1–14.

R6. Herzog, H.; Klein, A.; Bleckmann, H.; Holik, P.; Schmitz, S.; Siebke, G.; Tätzner, S.; Lacher, M.; Steltenkamp, S. μ-Biomimetic Flow-Sensors - Introducing Light-Guiding PDMS Structures into MEMS. *Bioinspiration and Biomimetics* **2015**, *10*, 36001.

R7. Kottapalli, A.G.P.; Asadnia, M.; Miao, J.M.; Barbastathis, G.; Triantafyllou, M.S. A Flexible Liquid Crystal Polymer MEMS Pressure Sensor Array for Fish-like Underwater Sensing. *Smart Mater. Struct.* **2012**, *21*.

R8. Shaikh, S.F.; Mazo-Mantilla, H.F.; Qaiser, N.; Khan, S.M.; Nassar, J.M.; Geraldi, N.R.; Duarte, C.M.; Hussain, M.M. Noninvasive Featherlight Wearable Compliant "Marine Skin": Standalone Multisensory System for Deep-Sea Environmental Monitoring. *Small* **2019**, *15*, 1–12.

R9. He, Q.; Zhang, W.; Sheng, T.; Gong, Z.; Dong, Z.; Zhang, D.; Jiang, Y. Flexible Conductivity-Temperature-Depth-Strain (CTDS) Sensor Based on a CNT/PDMS Bottom Electrode for Underwater Sensing. *Flex. Print. Electron.* **2022**, *7*.

Supplementary Movies

Movie

S1: Structure-electrostatic Multiphysics coupling simulations (Piezopotential distribution within piezoelectric film).

S2: FSI analysis for vibrating sphere location by DSHPS array (Position 1).

S3: FSI analysis for vibrating sphere location by DSHPS array (Position 2).

S4: FSI analysis for vibrating sphere location by DSHPS array (Position 3).

S5: FSI analysis for vibrating sphere location by DSHPS array (Position 4).

S6: FSI analysis for vibrating sphere location by DSHPS array (Position 5).
